# Genomics-based diversity analysis of *Vanilla* species using a *Vanilla planifolia* draft genome and Genotyping-By-Sequencing

**DOI:** 10.1038/s41598-019-40144-1

**Published:** 2019-03-04

**Authors:** Ying Hu, Marcio F. R. Resende, Aureliano Bombarely, Maria Brym, Elias Bassil, Alan H. Chambers

**Affiliations:** 1 Horticultural Sciences Department, University of Florida, Gainesville, FL USA; 2 School of Plant and Environmental Sciences, Virginia Polytechnic Institute and State University, Blacksburg, VA USA; 3 Department of Biosciences, Università degli Studi di Milano, Milan, Italy; 4 Tropical Research and Education Center, Horticultural Sciences Department, Homestead, FL USA

## Abstract

Demand for all-natural vanilla flavor is increasing, but its botanical source, *Vanilla planifolia*, faces critical challenges arising from a narrow germplasm base and supply limitations. Genomics tools are the key to overcoming these limitations by enabling advanced genetics and plant breeding for new cultivars with improved yield and quality. The objective of this work was to establish the genomic resources needed to facilitate analysis of diversity among *Vanilla* accessions and to provide a resource to analyze other *Vanilla* collections. A *V. planifolia* draft genome was assembled and used to identify 521,732 single nucleotide polymorphism (SNP) markers using Genotyping-By-Sequencing (GBS). The draft genome had a size of  2.20 Gb representing 97% of the estimated genome size. A filtered set of 5,082 SNPs was used to genotype a living collection of 112 *Vanilla* accessions from 23 species including native Florida species. Principal component analysis of the genetic distances, population structure, and the maternally inherited *rbcL* gene identified putative hybrids, misidentified accessions, significant diversity within *V. planifolia*, and evidence for 12 clusters that separate accessions by species. These results validate the efficiency of genomics-based tools to characterize and identify genetic diversity in *Vanilla* and provide a significant tool for genomics-assisted plant breeding.

## Introduction

Vanilla is the second most valuable spice with increasing global demand^1^. Vanilla is an extract produced from the cured seed capsules (commonly referred to as “beans”) of the vining orchid *Vanilla planifolia*. The extract is used as a premium ingredient in ice cream, chocolate, perfumes, pharmaceuticals, and other products^2^. *Vanilla* beans have been used for their valuable aroma since pre-colonial times by early Mesoamericans, including its use to improve the flavor of chocolate by the Aztecs and Mayans^3,4^. *Vanilla* spread globally from Mesoamerica starting in the late 1500s including introduction into Europe in 1739 and domestically from Florida to Puerto Rico sometime before 1900^5,6^. Today, clonal descendants, likely originating from these original introductions, are grown commercially. The result is an industry with heavy reliance on a limited genetic base. Improving the genetic diversity and horticultural performance of *Vanilla* is increasingly important as demand for natural ingredients like vanilla extract increases.

Historically, *Vanilla* production was limited to Mexico until supply constraints in the 1850s and 1860s pushed for expanded cultivation into other geographies. The demonstration of manual pollination in 1838 by Professor Charles Morren and an optimized, practical method in 1841 by a former slave named Edmond Albius enabled expanded commercial production beyond the native distribution^7,8^. Today, Madagascar dominates *Vanilla* production with Indonesia, Uganda, India, Comores, and Mexico all significantly contributing to global supply and with minor production in many other countries^9^. The United States imports more *Vanilla* beans than any other country (~1,500 to 2,000 metric tons annually), and produces high-value vanilla extract for export^10^. Cyclical supply deficits from major weather events, theft, poor quality beans, or geopolitical challenges have increased *Vanilla* bean price volatility. Recently, cured *Vanilla* beans were traded at over $600 per kilogram^11^. This price volatility can negatively impact growers, consumers, and the entire *Vanilla* supply chain.

Most vanilla extract comes from cured *V. planifolia* beans, but at least two other species including *V. x tahitensis* and *V. pompona* are also grown commercially on a more limited scale. The aroma profiles of each of these species vary, and these differences can be useful for various applications from specialty food ingredients to cosmetic uses. The standard of identity (CFR 169.175) for *Vanilla* bean extract includes only *V. planifolia* and *V. x tahitensis* for historical reasons^7^, though other species may have commercial relevance and favorable flavor profiles that could be used to improve the commercial species. Alternative sources of vanilla flavor include synthetic vanillin from paper and pulp mill byproducts, and petroleum-based synthesis^2^. Synthetic vanillin is important to meet the near insatiable demand for vanilla flavor, but industry trends based on consumer demands are increasingly favoring flavors from natural sources like the *Vanilla* bean.

*Vanilla* is pantropical, but the primary commercial species, *V. planifolia*, was first cultivated in Mesoamerica^3^. *V. planifolia* spread globally from Mesoamerica through the Caribbean islands and into Europe as early as 1510 with successful cultivation in England by 1807^8^. Vanilla was then introduced into Africa (1852), India (1835), and into today’s major commercial regions of Reunion Island (1793) and Madagascar (1870)^12^. Since the early 1900s, commercial *Vanilla* production in the United States has been confined to Hawaii and Puerto Rico. Hawaii has naturalized *Vanilla* introductions originating from Mexico, Tahiti, Samoa, and Fiji with some cultivated for commercial production^13^. Puerto Rico has both native species and *V. planifolia* that was introduced from Florida and has been in cultivation since the early 1900s^6^. The Hawaiian *Vanilla* industry is now mostly tourism-based, and Puerto Rican *Vanilla* cultivation declined in the 1950s with only remnants of escaped plants surviving to this day. Today, growers and industry representatives are expressing increasing interest in domestic *Vanilla* cultivation including expansion into other suitable environments like south Florida where both native species and naturalized *V. planifolia* are already growing. The four native species in Florida include *V. phaeantha* (“leafy *Vanilla*”), *V. barbellata* (“worm vine orchid”), *V. dilloniana* (“Dillon’s *Vanilla*”), and *V. mexicana* (“Fuchs’ orchid”)^14^. These native species are endangered and exist in protected areas, except for *V. dilloniana* that is thought to be extirpated. The origin of the naturalized *V. planifolia* population in Florida is currently unknown.

There are over 100 species of *Vanilla* distributed approximately from latitudes 27°N to 27°S around the world. Many of these species are morphologically distinct for various characteristics including the presence or absence of leaves, aromatic or non-aromatic beans, climbing habit, variation in flower coloration, leaf size, and leaf shape to name a few. Many *Vanilla* species share similar vegetative traits and require a mature, flowering specimen in order to confirm identity. Additionally, morphological traits can vary with maturity and environmental conditions making species-level identification challenging. Molecular markers have been used to assess the diversity of *Vanilla* accessions from both herbarium specimens and natural populations. Such marker studies include isozymes^15^, RAPDs^16–18^, AFLPs^4,19,20^, microsatellites^21–24^, single gene sequences (usually plastid-derived)^25–29^, or some combination of the above^30–35^. Major limitations of these approaches include a lack of reproducibility, poor transferability between labs, high relative cost, and low information density. Furthermore, many of the common markers used to identify *Vanilla* species cannot be used to identify hybrids, or to assign relationships beyond species-level resolution. The development of genomics-based platforms should overcome these limitations and would enable marker-trait associations for plant breeding research needed to develop improved *Vanilla* cultivars.

*Vanilla* in general has not benefited from strategic plant breeding and the use of segregating populations to identify marker-trait associations. Literature on *Vanilla* plant breeding is limited with references to older *Vanilla* breeding programs^12^. Previous research has described the results of a wide cross between *V. planifolia* and *V. aphylla* resulting in four hybrid progeny that may display delayed flower wilting^30,36^. *Vanilla* hybrids cultivated in Costa Rica and natural hybrids discovered in Puerto Rico have also been characterized^37,38^. Additionally, an AFLP-based *Vanilla* genetic linkage map using a *V. x tahitensis* and *V. pompona* population has been reported^20^. *Vanilla* plant breeding could greatly be enhanced through the development and application of genomics-based molecular markers like single nucleotide polymorphisms (SNPs). Indeed, many authors have discussed the extremely narrow genetic diversity of commercially grown *V. planifolia* originating from a few foundational clones^4,12,39–43^. There is no technical or biological limitation that would prevent the breeding of improved *Vanilla* cultivars with improved yield and quality.

The objective of this research was to test the ability of genomics to analyze diversity within a living collection of *Vanilla* accessions. The outcomes of this research include the assembly of a draft *Vanilla* genome, the development of genomic resources to rapidly genotype *Vanilla* species, the identification of hybrid accessions, the assignment of species for unknown samples, and the identification of species-specific SNP markers that could aid in *Vanilla* identification. These resources will support the development of new *Vanilla* cultivars to meet evolving industry and consumer needs, and support a domestic *Vanilla* industry.

## Results

### *Vanilla* accessions included in this study

One goal of this study was to assemble a living collection of diverse *Vanilla* species needed to develop genomics-based tools for diversity analysis. The collection sourced accessions from botanical gardens, online vendors, private collectors, and native species. Accessions were maintained at the Tropical Research and Education Center of the University of Florida, in Homestead, FL. All 112 accessions included in this study, including six replicates as sequencing controls, selected for genotyping-by-sequencing (GBS) analysis are described in Table 1. Species assignments have been updated based on the results described in this study.

**Table 1:** List of *Vanilla* accessions included in this study.

Identifier	Species	Source	Heterozygosity	BIC Group	Notes
AC101	*V. phaeantha*	Online vendor	0.0444	11	3
AC102	*V. siamensis*	Private collection	0.0313	9	
AC103	*V. pompona*	Private collection	0.0631	3	
AC104	*V. pompona*	Private collection	0.0584	3	3
AC105	*V. dilloniana* and unknown	Online vendor	0.0655	6	2
AC106	*V. planifolia*	Private collection	0.0457	5	1, 3
AC107	*V. pompona* and *V. phaeantha*	USDA	0.0600	3	2
AC108	*V. planifolia* and unknown	Online vendor	0.0401	5	1, 2, 3
AC109	*V. planifolia x V. phaeantha*	Botanical Garden	0.0498	11	2, 3
AC110	*V. pompona*	Private collection	0.0627	3	1, 3
AC111	*V. phalaenopsis*	Botanical Garden	0.0274	2	
AC112	*V. madagascariensis*	Botanical Garden	0.0348	2	
AC113	*V. planifolia*	Botanical Garden	0.0363	5	
AC114	*V. insignis*	Botanical Garden	0.0331	10	
AC115	*V. dilloniana*	Botanical Garden	0.0712	6	3
AC116	*V. phaeantha*	Botanical Garden	0.0404	11	
AC117	*V. planifolia*	Online vendor	0.0349	5	
AC118	*V. imperialis*	Botanical Garden	0.0631	2	3
AC119	*V. poitaei*	Online vendor	0.0409	6	3
AC120	*V. planifolia*	Botanical Garden	0.0322	5	
AC121	*V. aphylla*	Online vendor	0.0658	6	
AC122	*V. dilloniana*	Online vendor	0.0699	6	
AC123	*V. appendiculata*	Botanical Garden	0.0405	2	3
AC124	*V. phaeantha*	Botanical Garden	0.0433	11	1, 3
AC125	*V. imperialis*	Botanical Garden	0.0530	2	
AC126	*V. planifolia*	Naturalized species	0.0346	5	3
AC127	*V. roscheri*	Botanical Garden	0.0500	4	3
AC128	*V. roscheri*	Botanical Garden	0.0370	2	
AC129	*V. phaeantha* and unknown	Botanical Garden	0.0430	11	1, 2, 3
AC130	*V. planifolia x V. pompona*	Online vendor	0.0543	5	2, 3
AC131	*V. dilloniana*	Online vendor	0.0686	6	
AC132	*V. pompona x V. phaeantha*	Private collection	0.0788	7	1, 2, 3
AC133	*V. planifolia*	Online vendor	0.0363	5	1, 3
AC134	*V. dilloniana*	Online vendor	0.0673	6	1, 3
AC135	*V. phaeantha*	Native Species	0.0373	11	
AC136	*V. phaeantha*	Native Species	0.0368	11	
AC137	*V. phaeantha*	Native Species	0.0393	11	
AC138	*V. phaeantha*	Native Species	0.0409	11	
AC139	*V. barbellata*	Private collection	0.0428	4	3
AC140	*V. barbellata*	Private collection	0.0422	6	1
AC141	*V. roscheri*	Botanical Garden	0.0322	2	
AC142	*V. pompona* and *V. odorata*	Botanical Garden	0.0423	8	1, 2, 3
AC143	*V. palmarum* and unknown	Botanical Garden	0.0332	10	2
AC144	*V. imperialis*	Botanical Garden	0.0562	2	
AC145	*V. barbellata*	Native Species	0.0517	4	
AC146	*V. barbellata*	Native Species	0.0529	4	
AC147	*V. phaeantha x V. pompona*	Native Species	0.0557	7	2, 3
AC148	*V. barbellata* and unknown	Online vendor	0.0288	9	2
AC149	*V. barbellata*	Native Species	0.0503	4	
AC150	*V. claviculata*	Online vendor	0.0454	4	
AC151	*V. planifolia*	Private collection	0.0390	5	
AC152	*V. barbellata*	Private collection	0.0459	4	1
AC153	*V. pompona*	Botanical Garden	0.0584	3	
AC154	*V. griffithii*	Botanical Garden	0.0358	9	
AC155	*V. schwackeana*	Botanical Garden	0.0315	8	
AC156	*V. pompona*	Botanical Garden	0.0557	3	
AC157	*V. pompona* and *V. phaeantha*	Private collection	0.0823	7	2
AC158	*V. pompona* and *V. odorata*	Private collection	0.0454	8	2, 3, 5
AC159	*V. pompona*	Private collection	0.0638	3	5
AC160	*V. planifolia*	Private collection	0.0397	5	
AC161	*V. aphylla* and unknown	Private collection	0.0307	9	2
AC162	*V. imperialis*	Private collection	0.0768	2	
AC164	*V. planifolia*	Private collection	0.0362	5	5
AC165	*V. pompona* and *V. odorata*	Private collection	0.0393	8	2, 3, 5
AC166	*V. pompona*	Private collection	0.0573	3	
AC167	*V. pompona*	Private collection	0.0569	3	5
AC168	*V. planifolia*	Private collection	0.0352	5	3
AC169	*V. pompona*	Private collection	0.0570	3	
AC170	*V. planifolia x V. phaeantha*	Private collection	0.0502	11	2, 3, 5
AC171	*V. planifolia*	Botanical Garden	0.0357	5	
AC172	*V. imperialis*	Botanical Garden	0.0640	2	
AC173	*V. planifolia*	Private collection	0.0360	5	3
AC174	*V. planifolia*	Private collection	0.0385	5	1, 3
AC175	*V. pompona* and *V. odorata*	Private collection	0.0612	3	1, 2, 3
AC176	*V. planifolia*	Online vendor	0.0401	5	
AC177	*V. odorata*	Online vendor	0.0342	1	3
AC178	*V. planifolia*	Online vendor	0.0381	5	1, 3
AC179	*V. pompona* and unknown	Online vendor	0.0492	3	2
AC180	*V. planifolia*	Online vendor	0.0401	5	
AC181	*V. planifolia*	Online vendor	0.0361	5	1, 3
AC182	*V. dilloniana* and unknown	Private collection	0.0598	6	2
AC183	*V. pompona*	Private collection	0.0553	3	3
AC184	*V. planifolia*	Private collection	0.0365	5	3
AC185	*V. planifolia*	Private collection	0.0368	5	3
AC186	*V. planifolia*	Private collection	0.0405	5	3
AC187	*V. planifolia*	Private collection	0.0378	5	4 (AC173)
AC188	*V. planifolia*	Private collection	0.0354	5	4 (AC174)
AC189	*V. pompona x V. phaeantha*	Private collection	0.0781	7	2, 4 (AC132)
AC190	*V. pompona*	Private collection	0.0574	3	4 (AC104)
AC191	*V. mexicana*	Native Species	0.0375	9	3
AC192	*V. mexicana*	Native Species	0.0390	9	
AC193	*V. barbellata*	Private collection	0.0486	4	3, 5
AC194	*V. planifolia x V. phaeantha*	Botanical Garden	0.0517	11	2, 3
AC195	*V. planifolia*	Private collection	0.0343	5	3
AC196	*V. planifolia*	Private collection	0.0331	5	3
AC197	*V. planifolia*	Private collection	0.0342	5	3
AC198	*V. planifolia*	Private collection	0.0342	5	
AC199	*V. planifolia*	Private collection	0.0398	5	
AC200	*V. pompona*	Private collection	0.0643	3	
AC201	*V. planifolia*	Online vendor	0.0365	5	
AC202	*V. planifolia*	Private collection	0.0397	5	3, 5
AC203	*V. planifolia*	Private collection	0.0383	5	3, 5
AC204	*V. hartii*	Private collection	0.0376	10	3, 5
AC205	*V. x tahitensis*	Private collection	0.0389	5	3, 5
AC206	*V. x tahitensis*	Private collection	0.0367	5	3, 5
AC207	*V. odorata*	Private collection	0.0426	1	3, 5
AC208	*V. ensifolia*	Private collection	0.0305	8	3, 5
AC209	*V. planifolia*	Private collection	0.0364	5	3, 5
AC210	*V. odorata*	Private collection	0.0398	1	3, 5
AC211	*V. odorata* and unknown	Private collection	0.0299	1	2, 3, 5
AC212	*V. ensifolia*	Private collection	0.0328	8	3, 5
AC213	*V. odorata*	Private collection	0.0369	1	3, 5
AC214	*V. palmarum*	Private collection	0.0507	12	3, 5
AC215	*V. palmarum*	Private collection	0.0513	12	3, 5
AC216	*V. palmarum*	Private collection	0.0502	12	3, 5
AC217	*V. palmarum*	Private collection	0.0496	12	3, 5
AC218	*V. planifolia*	Private collection	0.0346	5	4 (AC173)
AC219	*V. planifolia*	Private collection	0.0345	5	4 (AC106)

Each accession was given a unique identifier from AC101 to AC219 (accession AC163 was dropped due to poor library QC results). Shown are the species assignments based on *rbcL* sequencing (as available) and GBS data, source of the material, calculated heterozygosity based on GBS, clustering group assignment (BIC Group), and notes. Notes are coded as follows: (1) previously misclassified accession, (2) probable hybrid based on GBS results, (3) accessions with *rbcL* sequence data provided in this study, (4) duplicate samples (with duplicate sample ID shown in parentheses), and (5) previously unknown species assignment. Predicted parents of hybrids are shown in place of a species assignment with maternal parent shown first when known.

### A draft genome for *V. planifolia*

A draft genome of accession AC173 was created to assist with read mapping and SNP calling. The estimated haploid genome size and heterozygosity were 1.13 Gb and 2.32%, respectively, based in the best model fit from GenomeScope (99.53% for 77 Kmer). The Kmer distribution did not show the two clear peaks that are usually present for an allotetraploid or hybrid. The genome size estimation using Kmers delivered a value that it is half of the values of previous estimations (1 C~2.26 Gb)^42^.

One paired end (insert size 300 bp) and three mate pair libraries (insert sizes 5, 8 and 10 Kb) were sequenced, delivering 45.90, 56.98, 53.04, and 47.36 Gb respectively (40X of paired ends and 139X of mate pairs). SOAPdenovo2 and Minia were used to assemble the sequencing data. For all of the assemblies, the SOAPdenovo2 assembly with a Kmer of 95 (VaplaK095A02) was preferred based on its assembly stats (see Methods for more details), and was selected for GBS read alignment. Gaps were filled in VaplaK095A02 to produce assembly Vapla0.1.1, and then contaminants and scaffolds less than 200 bp were filtered producing the final assembly Vapla0.1.4. Vapla0.1.4 is a highly fragmented assembly with a total size of 1.96 Gb (contigs) and 2.20 Gb (scaffolds) in agreement with published flow cytometry genome size estimations. Statistical results are summarized in Table 2.

**Table 2:** Statistical summary for contigs and scaffolds in the VaplaK095A02 and Vapla0.1.4 assemblies. Publicly available RNA-Seq data was used to test the quality of the draft genome assemblies.

Assembly	VaplaK095A02		Vapla0.1.4	
Assembly Statistics	Contigs	Scaffolds	Contigs	Scaffolds
Total assembly size (Gb)	1.20	2.34	1.96	2.20
Total assembled sequences	2,955,869	2,115,012	2,250,393	794,547
Longest sequence length (Kb)	54.03	626.54	129.70	630.91
Average sequence length (Kb)	0.40	1.12	0.87	2.77
N50 index (sequences)	445,497	11,865	47,789	9,596
L50 length (Kb)	0.60	41.10	9.62	53.33
% RNA-Seq Mapped	NA	87.1 ± 0.4	NA	96.7 ± 0.8
% BUSCO Completed	NA	8.6	NA	79.5
% BUSCO Duplicated	NA	1.2	NA	32.8
% BUSCO Fragmented	NA	16.7	NA	5

Two approaches were used to evaluate the completeness of the Vapla0.1.4 assembly gene space including (1) read mapping of three RNA-seq datasets from the National Center for Biotechnology Information (differentiated flower bud SRR1171644, placental laminae in mature pods of 6 months old *Vanilla* SRR1509374^44^, and leaf SRR1509356^44^), and (2) BUSCO^45^ analysis (Table 2). The high percentage of the reads of the different RNA-seq datasets that map to the Vapla0.1.4 reference indicates that the assembly draft may be capturing a high percentage of the gene space (>95%).

### SNP analysis

The GBS library produced 124,085,946 reads with the expected barcode and cut site overhang (99.99% of 124,089,684 total Illumina reads). The TASSEL 3 GBS pipeline identified 11,711,559 unique tags from high quality barcoded reads, of which 6,643,190 (56.72%) aligned to the VaplaK095A02 draft *Vanilla* genome. A total of 521,732 unfiltered SNPs were produced, which have 0.93–15.39 mean read depth and 33.24–94.21% missing rate for the 118 samples (Supplementary Table S1). 5,082 SNPs remained for downstream analyses after filtering for maximum missing rate (<30%), minor allele frequency (>10%), minimum read depth (>10), maximum read depth (<1000), maximum heterozygosity rate (<20%), and linkage disequilibrium (<0.2). The SNP analysis results are shown in Supplementary Fig. S1.

### Heterozygosity

The SNP analysis included calculations of heterozygosity for each accession as shown in Table 1. Heterozygosity as calculated by vcftools–het and ranged from 0.0274 for *V. phalaenopsis* AC111 to 0.0823 for AC157 from a private collection that is most likely a hybrid between *V. phaeantha* and *V. pompona*. Some species had an overall higher average rate of heterozygosity including *V. dilloniana*, *V. imperialis*, and *V. pompona*. Conversely, low average heterozygosity was calculated for *V. planifolia*, *V. odorata*, and *V. x tahitensis*. The native species ranged from lowest to highest heterozygosity for *V. mexicana* < *V. phaeantha* < *V. barbellata* < *V. dilloniana*, respectively.

### GBS-based diversity analysis

**Figure 1:**
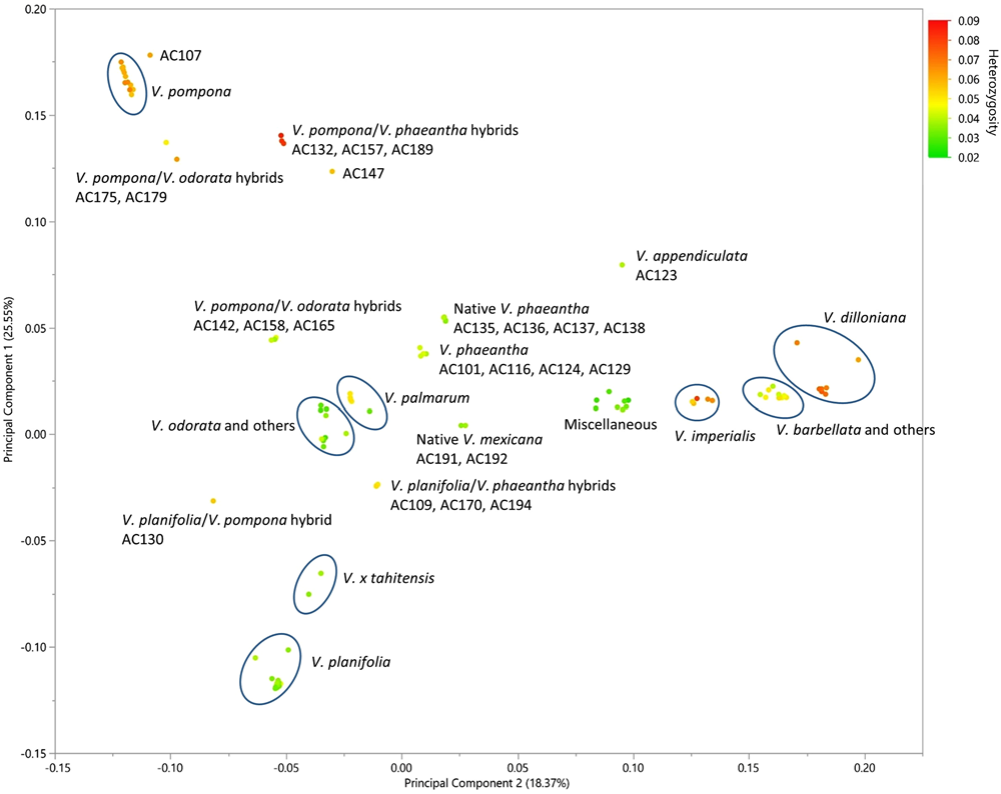
Plot of the first two principal components using 5,082 filtered SNPs. Individual accessions are shown by dots colored by heterozygosity rate with the highest heterozygosity shown in red and lowest shown in green. Species groupings are delineated by ovals surrounding groups of dots representing individual accessions.

Genetic diversity within the collection was assessed using the 5,082 filtered GBS SNPs. A plot of the first two principal components indicated that enough variation was captured within PCA1 (25.55%) and PCA2 (18.37%) to visually differentiate species (Fig. 1). Our PCA analysis grouped the accessions within distinct clusters representing the species *V. pompona*, *V. planifolia*, *V. imperialis*, *V. odorata*, *V. palmarum*, *V. barbellata*, *V. dilloniana*, *V. phaeantha*, *and V. mexicana*. Other species with only a few representatives clustered with *V. odorata*, *V. barbellata*, in a miscellaneous cluster, or were entirely separated as for the *V. appendiculata* (AC123) accession indicating that some accessions within this collection are genetically distinct. Exceptions to these trends were identified as probable hybrids as described below. Two probable *V. x tahitensis* accessions were located on the PCA plot between *V. planifolia* and *V. odorata*.

### Clustering and STRUCTURE analysis using filtered SNPs

**Figure 2:**
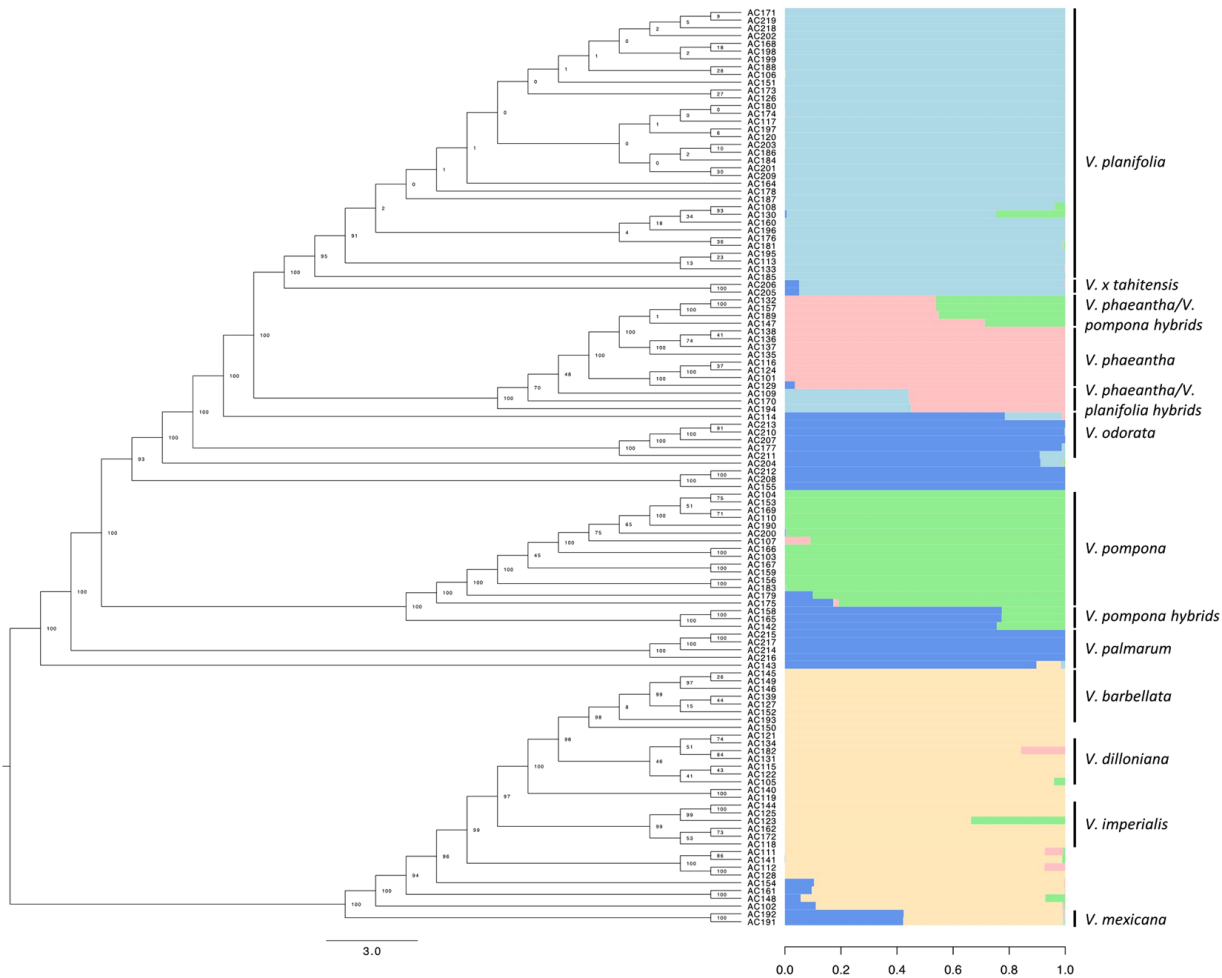
Cladogram and genetic structure of 112 accessions and six replicates included in this study. Phylogenetic tree constructed using SNP data with bootstrap percentage for 1,000 replicates is shown. Bayesian clustering (STRUCTURE, K = 5) of 118 accessions is shown on the right portion of the figure. The x-axis quantifies cluster membership, and the y-axis represents the different accessions. The order and position of accessions on the y-axis are consistent with those in the phylogenetic tree. General species groupings are shown alongside the STRUCTURE plot. STRUCTURE colors were assigned according to a best fit of K = 5 from STURCTURE HARVESTER.

Clustering, phylogenetic, and STRUCTURE analyses^46^ of the filtered SNPs revealed distinct relationships among accessions (Fig. 2). Discriminant analysis of principal components DAPC using the top 40 principal components yielded evidence for 12 clusters (BIC Groups, Table 1, Supplementary Fig. S2). *V. planifolia* accessions had lower bootstrap support than other species and were all in cluster 5. A few accessions had higher bootstrap support for separation from the majority of *V. planifolia* and included AC195, AC113 (variegated accession), AC133, and AC185. *V. x tahitensis* accessions AC205 and AC206 are also part of the *V. planifolia* cluster as would be expected, while all *V. odorata* (five accessions) are in cluster 1. Cluster 3 was mostly *V. pompona* with a few potential *V. pompona* hybrids including AC107, AC179, and AC175 while other putative *V. pompona* hybrids formed cluster eight (AC158, AC165, AC142).

The putative hybrid accessions and native *Vanilla* species were included in multiple clusters. The known *V. planifolia* x *V. pompona* hybrid AC130 was part of cluster 5 with the *V. planifolia* accessions. Hybrids between *V. phaeantha* and *V. pompona* formed cluster 7 with a few *V. pompona* and *V. phaeantha* accessions. Native and procured *V. phaeantha* accessions along with a group of *V. phaeantha*/*V. pompona* hybrids formed cluster 11. Cluster 4 included the native *V. barbellata* species and *V. claviculata*, and Cluster 6 included *V. dilloniana* and *V. poitaei*. Cluster 9 included *V. griffithii*, *V. aphylla*, *V siamensis*, and a *V. barbellata* accession along with the native *V. mexicana* accessions.

### Species identification using *rbcL* sequencing

**Figure 3:**
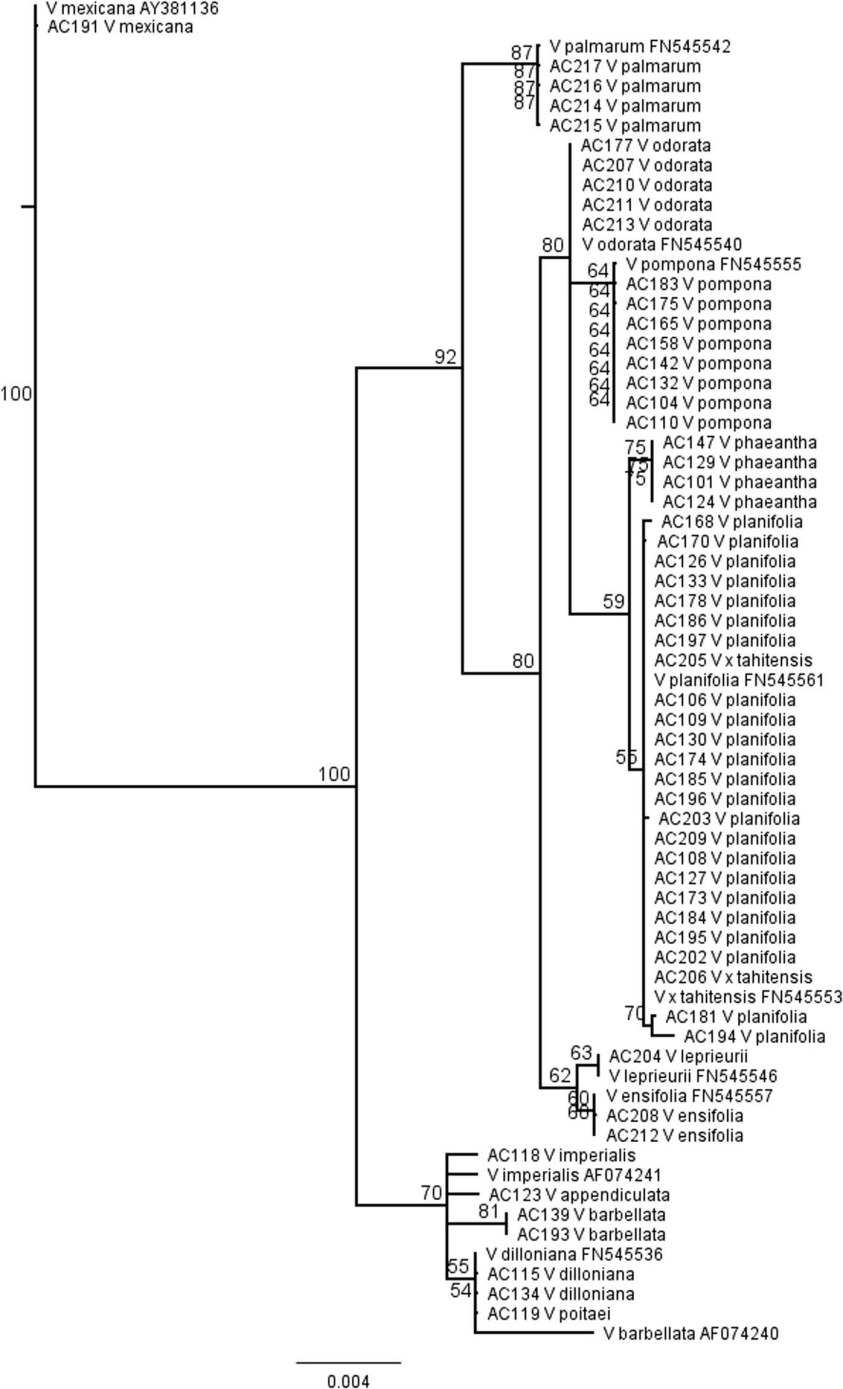
Phylogenetic tree based on ribulose-1,5-bisphosphate carboxylase/oxygenase large subunit (*rbcL*) partial locus sequencing for selected accessions in the study. Included are published *rbcL* sequences obtained from NCBI as indicated by accessions IDs. Bootstrap values for 100 clustering replicates are shown.

Single gene sequencing of *Vanilla* species is useful for identifying accessions at the species level^47^, because these sequences can be compared to publicly available data at bioinformatics repositories including the National Center for Biotechnology Information (ncbi.nlm.nih.gov). Single gene sequencing is also useful in the identification of maternal parents of potential hybrids, because many of the single gene sequences for genotyping *Vanilla* are from plastid-derived targets. Fifty-seven accessions were selected for single gene sequencing of the *rbcL* locus (Fig. 3). The results from this analysis are in close agreement with previously published sequences except for *V. barbellata* AF074240 where the reference sequence is more similar to *V. dilloniana* accessions than the *V. barbellata* accessions in this study. No *rbcL* reference sequences were available for *V. phaeantha*, *V. appendiculata*, or *V. poitaei* and were therefore not included in this analysis. GBS and *rbcL* sequencing results indicate that native *V. mexicana* is distinct from all other accessions.

### Diversity within *V. planifolia*

**Figure 4:**
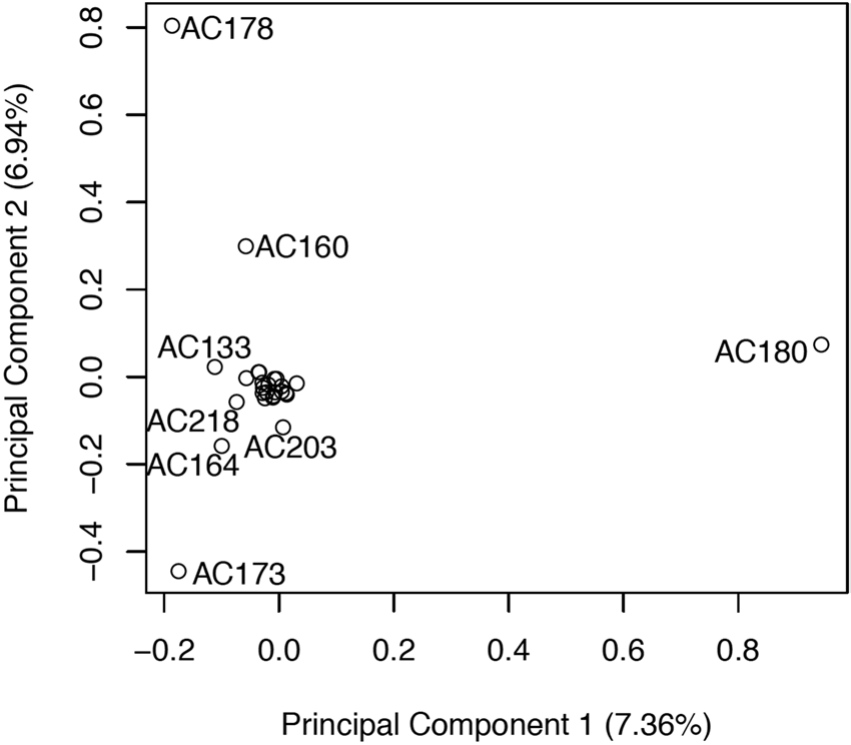
PCA plot showing diversity within 27 *V. planifolia* accessions using 565 filtered SNPs. Diverse accessions AC108, AC181, and AC185 were excluded to improve resolution of the remaining accessions. Distinct accessions are labeled by accession number.

*V. planifolia* is the major commercial species for the genus, and thus diversity within this species is especially valuable for future plant breeding research. SNPs specifically selected for *V. planifolia* analysis would be expected to vary from the 5,082 SNPs selected to analyze diversity among many species based on the various filtering criteria imposed. Diversity within *V. planifolia* was therefore analyzed separately from the other species. The results are shown for 27 accessions in Fig. 4. There were 565 SNPs identified and used for this analysis. *V. planifolia* accessions AC181 and AC185 are more distantly related than the other *V. planifolia* accessions and were excluded to improve the resolution of the remaining accessions in Fig. 4.

### Fst analysis

**Figure 5:**
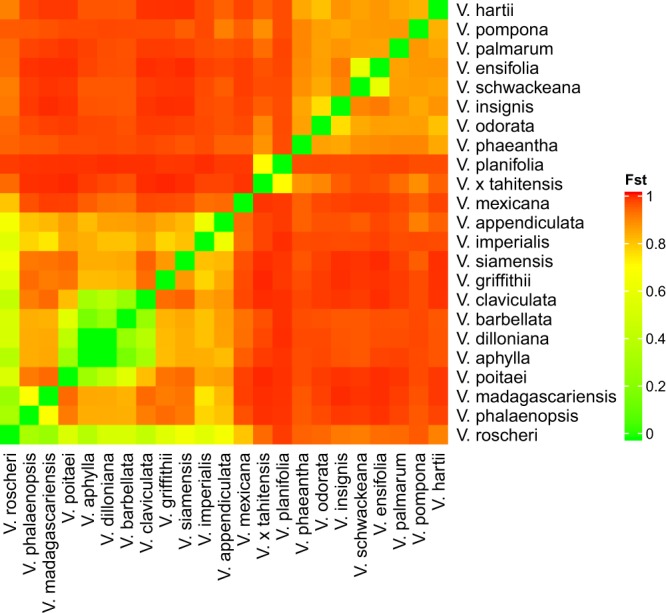
Heatmap illustrating pairwise Fst values among 23 *Vanilla* species. High Fst values are shown in red and low Fst values are shown in green.

Fst reflects the population differentiation due to genetic structure. High Fst values indicate a considerable degree of differentiation among populations. Distinct groups were identified from Fst analysis of the *Vanilla* collection (Fig. 5). One group includes *V. phaeantha*, *V. schwackeana*, *V. ensifolia*, *V. odorata*, *V. insignis*, *V. pompona*, *V. palmarum*, and *V. hartii* which share moderate pairwise Fst values. A second group includes *V. aphylla*, *V. roscheri*, *V. barbellata*, *V. claviculata*, *V. dilloniana*, *V. poitaei*, *V. griffithii*, *V. siamensis*, *V. imperialis*, *V. appendiculata*, *V. madagascariensis*, and *V. phalaenopsis* which also share low to moderate pairwise Fst values. These two groups share lower Fst, indicating that lower genetic differences exist among accessions in each group. Additionally, *V. planifolia* and *V. x tahitensis* share moderate pairwise Fst (0.704), meaning that these two have somewhat dissimilar genetic structure. The phylogenetic tree and STRUCTURE analysis also supported the similar grouping of *V. planifolia* and *V. x tahitensis*.

Fst analysis also separated most of the leafless species by low Fst values when compared to each other and high values when compared to *V. planifolia*. This pattern includes accessions for *V. aphylla, V. roscheri*, *V. barbellata*, *V. claviculata*, *V. dilloniana*, and *V. poitaei*. The exceptions include *V. claviculata* compared to *V. poitaei*, and *V. madagascariensis* that is leafless yet has more moderate Fst values compared to the other leafless species.

### Kinship analysis

**Figure 6:**
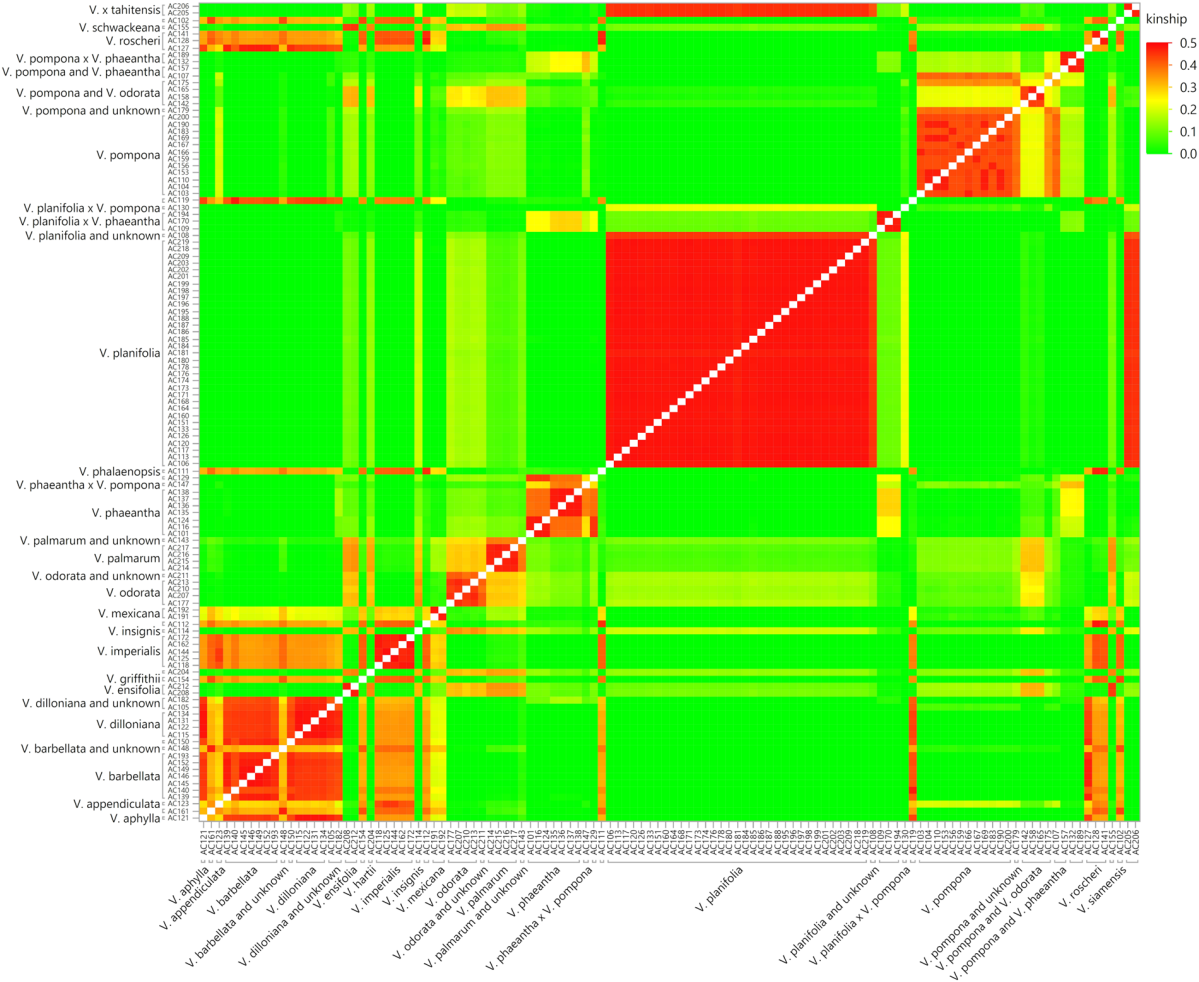
Kinship heatmap of 112 accessions and six replicates in this study. Accessions are grouped by species as assigned by *rbcL* sequencing (as available) and by GBS results. Lower kinship values are shown in green, moderate values in yellow, and higher values are shown in red.

Kinship analysis was conducted to test the relationship among individuals (Fig. 6). As expected, the species with multiple accessions (for example, *V. planifolia* and *V. pompona*) showed high kinship values among individuals of their respective species. *V. x tahitensis* has high kinship with *V. planifolia* as expected, and moderate kinship with *V. odorata*. Probable hybrid accessions can be visually identified by species assignment and contrasting kinship values compared to the majority of accessions in that species. The most diverse species including the leafless species tend to have higher kinship values with each other than to leafy species including *V. planifolia* and *V. pompona*.

### Identification of probable hybrids

Genomics-based analysis of the *Vanilla* collection enabled the identification of probable hybrids. One accession, AC130, is a *V. planifolia* (maternal parent) x *V. pompona* (paternal parent) hybrid confirmed by sequencing both the partial *rbcL* gene and by cloning and sequencing ITS amplicons from this accession (Supplementary Fig. S3). The hybrid nature of this accession is confirmed as shown in the STRUCTURE analysis including roughly three quarters *V. planifolia* alleles and one quarter *V. pompona* alleles.

Other potential hybrids were also identified by interpreting the combined results from the clustering and STRUCTURE analyses. These included accessions AC132 and AC157 that were received as *V. odorata* and *V. pompona*, respectively, but both contain markers for *V. pompona* and *V. phaeantha*. The maternal parent of AC132 was confirmed to be *V. pompona* by *rbcL* sequencing in agreement with the GBS results. AC189 is a duplicate sample of AC132 and the GBS results are consistent between the two samples. Both of the preceding accessions were from a single source, and could actually be cuttings from one original sample. The GBS results for AC147 provide support that this accession is a hybrid between *V. phaeantha* and *V. pompona*, but in contrast to AC132 and AC157, the maternal parent of AC147 was *V. phaeantha* as confirmed by *rbcL* sequencing. AC147 is unique as a probable hybrid because it was collected in southern Florida on protected land. Accessions AC132 and AC157 are separated on the PCA plot, and are unlikely to be from the same original clone.

AC109, AC170, and AC194 are all probable hybrids between *V. planifolia* and *V. phaeantha*. Each has *V. planifolia* as the maternal parent as confirmed by *rbcL* sequencing, are similarly located on the PCA plot, and have higher average heterozygosity (0.051) than *V. planifolia* (0.036). Each of these three accessions came from a different source (one private collector and two different botanical gardens). *V. pompona* was shown as the maternal parent of putative hybrids AC142 (sourced from a botanical garden), AC158 and AC165 (private collection), and AC175 (a separate private collection) yet all showed hybrid characteristics from the STRUCTURE analysis potentially with *V. odorata* in their ancestry. AC175 was part of cluster 3 while AC142, AC158, and AC165 formed cluster 8 with *V. ensifolia* and *V. hartii*. All potential hybrids are noted in Table 1.

### Misclassified accessions

There were 15 obviously misclassified accessions in this study, but only species with multiple accessions could be used to create consensus species assignments based on GBS and single gene sequencing. AC140 and AC152 were received as *V. dilloniana*, but are actually *V. barbellata*. AC181, AC133, and AC178 were received as *V. imperialis*, *V. mexicana*, and *V. pompona*, respectively, and are *V. planifolia*. AC132, AC110, AC142, and AC175 were received as *V. odorata*, *V. planifolia*, *V. planifolia*, and *V. planifolia*, respectively, and are *V. pompona*. AC134 was received as *V. phaeantha* and is *V. dilloniana*. AC124 and AC129 were received from two different botanical gardens as *V. x tahitensis* and are actually both *V. phaeantha*. AC106, AC108, and AC175 were also received as *V. x tahitensis*, but in the absence of *V. odorata* alleles were reclassified as *V. planifolia* according to their genotypes.

Other accessions may be misclassified, but lack sufficient supporting information to confidently reassign a species designation. For example, AC123 that was received as *V. appendiculata*, but single gene sequencing and GBS results both closely match *V. imperialis*. This could be biologically relevant, or an incorrect species assignment. Overall, the misclassified accessions were not limited to a single germplasm source, but were received from multiple botanical gardens, online vendors, and private collections.

### Species-specific SNPs

Species-specific, diagnostic molecular markers would be advantageous for quickly identifying species in new collections, and also for confirming hybrid progeny when breeding. The greatest limitations to validating species-specific SNPs include sampling enough diversity within a species and across relevant species to obtain high confidence for marker specificity. Towards developing species-specific SNPs, the 521,732 SNPs from this study were screened for those that could be species-specific. The results are reported in Supplementary Data S1. *V. planifolia* had 1,611 species-specific SNPs, and *V. pompona* had 3,230. The other species had values ranging from 227 for *V. aphylla* to 6,187 identified for *V. palmarum*.

## Discussion

The primary objective of this study was to examine the utility of genomics-based diversity analysis to characterize a living *Vanilla* collection. The collection included 112 accessions from 23 species obtained from botanical gardens, private collections, online vendors, and collected from natural areas in southern Florida as part of conservation research. We developed a draft *Vanilla* genome as a reference for GBS analysis of the living collection. GBS yielded 5,082 filtered SNPs resulting in the largest genomics dataset for *Vanilla* to date, and the first application of genomics-based diversity analysis in this genus. The increased resolution among accessions due to increased marker numbers and reduced cost per data point accelerated the discovery of potential hybrids, identified misclassified accessions, and demonstrated the suitability of these methods to analyze diversity across and within *Vanilla* species.

### A draft *Vanilla* genome for diversity analysis

We created a draft *V. planifolia* draft genome assembly to facilitate read mapping and SNP calling. The assembly proved its utility for the development of genetic markers and the study of the population structure in a biodiversity panel in spite of being highly fragmented. The *V. planifolia* genome size was previously reported as 2.26 +/− 0.05 Gb using flow cytometry^48^, being twice the genome size that was estimated by Kmer distributions. This could indicate that either previous flow cytometry values overestimated genome size, or that *V. planifolia* is an autopolyploid (2n = 4x = 24) with a haploid genome size of ~2.26 Gb. Using flow cytometry to estimate the genome size of orchid species is especially challenging due to endoreduplication^48^.

Leveraging the draft genome simplified SNP calling, but also presented a few limitations. For example, the number of mapped reads is expected to decrease as diversity increases possibly leading to the exclusion of SNPs that could be relevant to studies with other *Vanilla* species. Still, having obtained over 5,000 filtered SNPs for diversity analysis across the 23 species was more than sufficient to meet the study objectives. In the future, the results from the draft genome could be further developed to generate a *V. planifolia* reference genome that would capture a greater proportion of the genic space, and would enable gene discovery.

### Diversity analysis

The level of similarity among *V. planifolia* accessions, even those collected from disparate sources, is not surprising considering the general consensus that genetic diversity in *V. planifolia* is limited. This is probably due to the ease of propagation by cuttings and the worldwide distribution of a few foundational clones. Still, evidence of diversity among *V. planifolia* accessions was found in this study and includes a few accessions with only limited source information. All results from *rbcL* sequencing matched expectations from the published literature except for misclassified accessions^29^. Some *V. planifolia* accessions were genetically distinct. For example, AC133 was originally labeled as *V. mexicana*, but was confirmed to be *V. planifolia* by *rbcL* sequencing and was distinguished from the majority of the *V. planifolia* accessions based on the GBS results. AC185 was obtained from a commercial grower and described as a plant with unique morphology. The GBS results support the hypothesis that this accession is genetically distinct compared to other *V. planifolia* accessions including other types from the same source (AC184 and AC186). These results suggest that expanded sampling using genomics-based analysis methods could uncover hidden diversity even within *V. planifolia*. Uncovering genomics-based diversity could be a particularly useful tool when selecting parents for a *Vanilla* breeding program.

*V. x tahitensis* is a commercial species that can be sold with the “vanilla bean” label in the US and European markets. While *V. planifolia* commands much more market share, *V. x tahitensis* has a unique flavor profile and interesting agronomic characteristics including non-dehiscence^49,50^. It is therefore an important species within the genus and warrants additional genomics-based research. Previous work has shown a close genetic relationship between the *V. planifolia* and *V. x tahitensis*^4,16,18,33^, but definitive data (especially at the genomics level) supporting the origins of *V. x tahitensis* is still lacking*. V. x tahitensis* is not easily differentiated from *V. planifolia* using single gene sequencing as the two are closely related, but use of genomics-based molecular markers should be able to efficiently identify alleles from both *V. planifolia* and *V. odorata* in *V. x tahitensis*.

The *V. planifolia* and *V. odorata* hybrid origin hypothesis of *V. x tahitensis* could easily be tested using a genomics-based approach^33^. Accessions AC205 and AC206 are probably true *V. x tahitensis* clones based on PCS, STRUCTURE, and the current hybrid origin theory. The STRUCTURE results for these accessions show a high level of *V. planifolia* ancestry and only a low level of *V. odorata* ancestry. Both of these accessions were donated from a private collection as unknown species. The accessions were artificially propagated from material most likely collected in Belize where *V. x tahitensis* could be endemic^51^. Further genomics research is needed to characterize additional *V. x tahitensis* accessions in order to identify its origins and unlock the traits that make this hybrid unique from agronomic and sensory perspectives.

### Native *Vanilla* species

The four native Florida *Vanilla* species *V. phaeantha*, *V. barbellata*, *V. dilloniana*, and *V. mexicana* are all endangered. Conservation of these native species including collection, propagation, and reintroduction can be aided by diversity analysis. The native *V. phaeantha* species formed its own branch when clustering using the GBS data. This suggests some level of genetic diversity compared to *V. phaeantha* from other geographies. The identification of a potential hybrid between *V. phaeantha* and *V. pompona* (AC147) in a natural setting was unexpected. There are no known established populations of *V. pompona* in southern Florida, but *V. pompona* is common in private collections. The origin of this hybrid could exemplify the ease with which *Vanilla* species hybridize and establish in natural areas. This would suggest that hybrids like *V*. x *tahitensis* and AC147 could be more common than currently presumed.

*V. barbellata* and *V. dilloniana* are difficult to identify morphologically due to their shared leafless morphology and overlapping growing regions. GBS-based species identification could be a powerful tool for distinguishing these species as shown in this study. In contrast, *V. mexicana* appears to be morphologically distinct among *Vanilla* species, and also by single gene and GBS analysis. Future conservation work for each of these species should focus on identifying and maintaining as much diversity as can be captured prior to habitat destruction. The results from this study show the potential of GBS to support conservation efforts for these species.

### Analysis of hybrids and misidentified accessions

We identified 21 potential hybrids out of 112 total accessions (18.8%). The actual occurrence of hybrid accessions is probably underestimated in other collections, because the majority of common molecular markers used in *Vanilla* are from plastid-derived genes. This common strategy would only identify the maternal parent of a potential hybrid. The value of the GBS approach to characterize diversity in a *Vanilla* collection has also been shown to putatively identify the paternal parent of hybrid accessions more efficiently and cost effectively than other methods.

Approximately 13% of accessions in this study were confirmed to be misidentified based on consensus, and this is probably an underestimate. The consensus approach can identify the species of unknown accessions as long as verified species are included in the database. Some species in the collection are represented by one or a few accessions, thereby reducing confidence in assigning and re-assigning species identities. Missing data can also cause artifacts in the clustering analysis. Therefore, the results for accessions with limited sample numbers should be cautiously interpreted. The genomics-based characterization of *Vanilla* collections, including those at botanical gardens, would greatly benefit the *Vanilla* community, especially when sharing germplasm.

### Future work

Species-specific molecular markers would be useful for identifying unknown *Vanilla* accessions, and for confirming parentage of interspecific hybrid progeny. Additionally, a fewer number of informative SNPs could be used to differentiate accessions at reduced cost. Unknown and misclassified accessions are common in *Vanilla*, because species can look very similar and morphological traits (including leaf shape) can vary during development and across environments. Flower morphology often supports a species designation, but flowering can be an infrequent and unreliable event. One limitation of our approach for identifying diagnostic markers relies on the use of the *V. planifolia* draft genome for read mapping and SNP calling. While this approach has several advantages, it is expected that diverse species will have fewer mapped reads and therefore fewer SNPs called. This could favor SNP calls across conserved loci and result in the identification of fewer species-specific markers for distantly related accessions. Ultimately, species-specific SNPs like those included in Supplementary Data S1 will have to be identified and validated based on specific research objectives.

In conclusion, the development of a draft *V. planifolia* genome and genotyping a living collection of *Vanilla* species enabled diversity analysis, hybrid identification, and the designation of species assignments. The benefits of using GBS SNP data compared to other molecular markers include transferability between labs, ease of marker development, reduced genotyping costs, and increased reproducibility. Results from this work can easily be expanded to other *Vanilla* collections. Anticipated benefits would support breeding programs and the creation of improved *Vanilla* cultivars needed to meet cyclical supply challenges and prepare *Vanilla* to enter a modern era of cultivar development.

## Methods

### *Vanilla* plant accessions

*Vanilla* cuttings were obtained from domestic botanical gardens, local orchid enthusiasts, online vendors, and international collaborators to establish a living collection at the University of Florida’s Tropical Research and Education Center in Homestead, Florida. The collection comprises 112 accessions from 23 species, and also included accessions with unknown species designations. The list of *Vanilla* accessions used in this study is summarized in Table 1.

Native Florida *Vanilla* species were collected from southern Florida county and state parks under research permits working with park biologists following state regulations. *V. barbellata* and *V. phaeantha* were propagated by cuttings. *V. mexicana* leaves were sampled directly from different individuals in the natural setting, because this species is difficult to propagate by cuttings. *V. dilloniana* is extremely rare if not extinct in natural areas, and was therefore obtained through online vendors and local orchid enthusiasts. Collection sites cannot be disclosed due to permitting restrictions instituted to protect endangered species.

### Plant maintenance

Cuttings were established on raised beds with 16 cm of mulch in a shade house under 50% filtered light. Beds were 18 m long and 1.8 m wide. Individual trellises were spaced 1.2 m apart and included a central post, a column of wire mesh as a climbing substrate, and horizontal wooden post for vine support at 1.2 m from the ground. Supplemental irrigation was supplied through drip lines during the dry season. Average temperatures in Homestead, Florida vary between 18 °C in the winter months to 27 °C in the summer. Average rainfall is ~4.4 cm per month in the dry season (November to March), and up to ~20.6 cm on average per month during the peak of the rainy season (June to September) (Florida Automated Weather Network, http://fawn.ifas.ufl.edu)^52^.

### DNA extraction

DNA extraction was performed using a modified CTAB method. 1 gram (fresh weight) of mature leaf tissue was ground to a fine power in liquid nitrogen, mixed with 3 ml of DNA extraction buffer (2% CTAB, 1.4 M NaCl, 100 mM Tris, 2 mM EDTA, 1% β-mercaptoethanol, pH 8.0) in a 15 ml conical tube, vortexed to completely disperse the tissue, and then incubated at 65 °C for 10 minutes. Samples were then cooled to room temperature for 3–5 minutes and 3 ml chloroform:isoamyl alcohol (24:1 v/v) was added to each sample, vortexed, and centrifuged for 10 minutes at 18,000 × g. The supernatant was transferred to a fresh tube to precipitate DNA using two volumes of 95% ethanol. Samples were centrifuged at 10,000 × g for 10 minutes to pellet DNA, aspirated, washed once with one ml of 70% ethanol, centrifuged at 10,000 × g for 2 minutes, aspirated, and finally resuspended in 75 µl molecular biology grade water.

### A draft *V. planifolia* genome

A draft *V. planifolia* genome was created to facilitate read mapping and SNP calling. *V. planifolia* accession AC173 was selected for sequencing, and is a clone of the same accession used for a previously published transcriptome^44^. DNA was extracted as above, and submitted to the Duke GCB Sequencing Core for library preparation, and sequenced on two lanes of an Illumina HiSeq 4000 (paired-end 150 bp). Libraries with four insert sizes including mate-pairs (300 bp, 5–7 kb, 8–10 kb, and 10–12 kb) were used to create the draft genome. Adapters, low quality extremes (qscore < 30) and reads shorter than 50 bp were removed using Fastq-mcf from the Ea-utils package version 1.1.2-537^53^. PCR duplications were filtered using Prinseq version 0.20.4^54^. Reads were corrected using Musket version 1.1^55^. Genome size and heterozygosity were estimated analyzing the Kmer distribution^56^ using Jellyfish version 2.2.6^57^ and GenomeScope (web accessed on 2018-02-07)^58^ for the Kmers 31, 55, and 77. The reads were assembled using two different assemblers: Minia version 3, git commit efef7c7^59^, and SOAPdenovo2 version r240^60^. The assemblers were run with the following list of different kmers: 17, 21, 25, 31, 39, 47, 51, 53, 55, 57, 59, 61, 63, 71, 79, 87, 95, 103, 119 and 123. The assemblies were evaluated based on minimum difference between assembly size and genome estimated size, longest contig sequence, N90/L90, and N50/L50. The assembly with the highest rank across these parameters was selected for scaffolding and gap filling with the SOAPdenovo2 package version r240^60^.

The evaluation of the completeness of the assembly was performed using two approaches. First, three different RNA-seq datasets were downloaded from the NCBI SRA database including (1) SRR1171644: differentiated flower bud, (2) SRR1509374: placental laminae in six weeks initial pods, and (3) SRR1509356: leaves. Reads were processed with Fastq-mcf as described previously. Reads were mapped to the assembly version VaplaK095A02 using Hisat2 version 2.1.0^61^ with the default parameters. Second, BUSCO version 3.0.2^45^ was run on the assembly VaplaK095A02 with the default parameters.

Assemblies were run on the Cascades server at the Advance Research Computer center (https://www.arc.vt.edu/computing/cascades/) using a Largemem node. Assembly gene space evaluations were run on a Ubuntu server (Linux 4.4.0-97) with 64 threads, 128 Gb of RAM memory and 2 Tb of hard drive.

### Sequencing the *Vanilla* collection

Library preparation and sequencing for Genotyping-By-Sequencing (GBS) was performed at the University of Minnesota Genomics Center on a NextSeq 1 × 150 bp using 300–744 bp size selection with dual restriction enzymes BamHI and NsiI. A pilot study using eight *Vanilla* accessions found that 1 M reads per sample was sufficient to obtain an informative number of SNPs (data not shown). Therefore, 1 M reads per sample was selected for the 118 samples in this study including 112 accessions and 6 duplicates as sequencing controls.

### SNP calling

The Tassel 3 GBS pipeline^62^ was used to call SNPs from the sequenced GBS library using a reference draft *V. planifolia* genome. Quality filtering was performed primarily using build-in functions in VCFtools^63^. The minor allele frequency filter was set to 0.1 and minimum locus coverage was set to retain SNPs that are covered in at least 70% of the individuals. The minimum read depth and maximum read depth were set to 10 and 1000, respectively. The heterozygosity rate was set to 0.2 to eliminate SNPs with high amount of heterozygous calls resulting from alignment of paralogs and duplicate sequences. The SNPs only existing in one species were identified as species-specific SNPs from the total 521,732 SNPs.

### Phylogenetic analysis

The filtered VCF files were converted to Phylip format by concatenating the SNPs with PGDSpider v.2.0.9.0^64^ with IUPAC ambiguity codes for polymorphic data. The maximum likelihood (ML) phylogenetic tree with 1000 rapid bootstrap inference was constructed by using RAxML v.8.2.12^65^. The analysis was run using an ascertainment bias correction (ASC) for the data set containing only concatenated informative SNP positions, and a general time-reversible substitution model accounting for among-site rate heterogeneity (ASC_GTRGAMMA model). The RAxML results were graphically visualized in FigTree v.1.4.3 (downloaded from http://tree.bio.ed.ac.uk/software/figtree/).

### Population STRUCTURE

The population structure was investigated to identify clusters of genetically related individuals using the Bayesian clustering method implemented in STRUCTURE, v.2.3.4^46^. Ten independent STRUCTURE runs were performed for each of K = 2–16 (K = number of genetic clusters) with a burn-in of 10000 and 20000 iterations. The number of clusters that best fit the observed genotype data was inferred by examining the average and standard deviation (SD) of the natural log probability of each model^66^ using the online version of STRUCTURE HARVESTER^67^. Additionally, the R package SNPRelate^68^ was used to perform principal component analysis (PCA) and the R package adegenet^69^ was used to perform discriminant analysis of principal components (DAPC). Loci were trimmed to linkage disequilibrium (LD < 0.2) using the command “snpgdsLDpruning”. DAPC was performed on all individual genotypes by using the successive K-means approach, implemented by the find.clusters function, to identify the optimal number of groups based on Bayesian Information Criterion (BIC).

To investigate the diversity of *V. planifolia* further, the *V. planifolia* only PCA was performed on 27 accessions where three diverse *V. planifolia* lines (AC108, AC181, and AC185) identified from structure analysis were excluded. Pairwise Fst were calculated with the Weir and Cockerham formula^70^ with the SNPRelate package^68^.

### *rbcL* sequencing

Partial sequencing of the *rbcL* gene^26^ was preferentially used to confirm species identifications of selected accessions and to identify the maternal parent of probable hybrids. Primers *rbcL* forward 5′ CTTCACAAGCAGCAGCTAGTTC 3′ and *rbcL* reverse 5′ ATGTCACCACAAACAGAAAC 3′ were used to amplify an ~1,300 bp fragment of the *rbcL* gene in 25 µl reactions using Phusion High-Fidelity polymerase (F531, Thermo Scientific) according to the manufacturer’s instructions. PCR amplification was conducted using an Applied Biosystems SimpliAmp Thermal Cycler with the following program: initial denaturation 5 minutes at 95 °C followed by 30 amplification cycles of 30 seconds at 95 °C, 30 seconds at 55 °C, and 60 seconds at 72 °C, with a final 10 minute extension at 72 °C. Sanger Sequencing was conducted at GENEWIZ (GENEWIZ, South Plainfield, NJ) using *rbcL* forward as the sequencing primer. Sequences were verified against published *Vanilla rbcL* sequences from NCBI (ncbi.nlm.nih.gov) using the default settings for MUSCLE alignment^71^ and Geneious Tree Builder within the Geneious Software package (Geneious version 11.1, Newark, NJ). Selected NCBI sequences included *V. mexicana* (AY381136), *V. dilloniana* (FN545536*), V. barbellata* (AF074240), *V. imperialis* (AF074241), *V. palmarum* (FN545542), *V. leprieurii* (FN545546), *V. ensifolia* (FN545557), *V. x tahitensis* (FN545553), *V. planifolia* (FN545561), *V. pompona* (FN545555), and *V. odorata* (FN545540).

Accession AC130 was suspected of being a *V. planifolia* x *V. pompona* hybrid based on the prevalence of this genotype where it was sourced in Costa Rica^37^. Its hybrid status was confirmed by PCR amplification (as above), cloning (TOPO TA Cloning Kit, Thermo Fisher), and sequencing (as above) the ITS partial locus using forward primer 5′ TATGCTTAAAYTCAGCGGGT 3′ and reverse 5′ AACAAGGTTTCCGTAGGTGA 3′ with T7 as the sequencing primer^34,37^.

## Supplementary information

Supplementary Information

Supplementary Table S2

Supplementary Data S1

## Data Availability

*rbcL* and ITS sequences for the accessions in this study are deposited at NCBI under the Genbank IDs listed in Supplementary Table S3. The Whole Genome Shotgun project including raw reads and the draft genome have been deposited at DDBJ/ENA/GenBank under BioProject PRJNA507095. The genome version described in this paper is SDXO01000000. The GBS datasets are deposited at NCBI under BioProject PRJNA507246.

## References

[1] Gallage, N. J. & Møller, B. L. Vanilla: The most polular flavour in *Biotechnology of Natural Products* (eds Schwab, W., Lange, B. M. & Wüst, M.) 3–24 (Springer, 2018).

[2] Gallage NJ, Møller BL (2015). Vanillin–bioconversion and bioengineering of the most popular plant flavor and its de novo biosynthesis in the vanilla orchid. Mol. Plant.

[3] Bruman H (1948). The culture history of Mexican vanilla. Hisp. Amer. Hist.Rev..

[4] Lubinsky P, Bory S, Hernandez J, Kim SC, Gomez-Pompa A (2008). Origins and dispersal of cultivated vanilla (Vanilla planifolia jacks. [Orchidaceae]). Econ. Bot..

[5] Fouche JG, Jouve L (1999). Vanilla planifolia: history, botany and culture in Reunion island. Agronomie.

[6] Childers, N. F. *Vanilla culture in Puerto Ric*o. Vol. 28 (US Department ofAgriculture, 1948).

[7] Berenstein N (2016). Making a global sensation: Vanilla flavor, synthetic chemistry, and the meanings of purity. Hist. Sci..

[8] Childers, N., Cibes, H. & Hernandez-Medina, E. Vanilla-the orchid of commerce in *The Orchids* (ed. Withner, C.) 477–508 (The Ronald Press Company, 1959).

[9] Divakaran, M., Nirmal-Babu, K. & Grisoni, M. Biotechnological applications in vanilla in *Vanilla* (eds Odoux, E. and Grisoni, M.) 51–73 *(CRC Press*, 2010).

[10] FAO. Vanilla: Post-harvest Operations (2009).

[11] Pilling, D. The real price of Madagascar’s vanilla boom. *FInancial Times*, https://www.ft.com/content/02042190-65bc-11e8-90c2-9563a0613e56 (June 5^th^, 2018).

[12] Bory S, Grisoni M, Duval MF, Besse P (2008). Biodiversity and preservation of vanilla: present state of knowledge. Genet. Resourc. Crop Evol..

[13] Conter, F. E. Vanilla cultivation in Hawaii. *Press bulletin (Hawaii Agricultural Experiment Station); no. 6* (1903).

[14] Chambers, A. H. Establishing Vanilla Production and a Vanilla Breeding Program in the Southern United States. In *Handbook of Vanilla Science and Technology* (eds Havkin-Frenkel, D. & Belanger F. C.) 165–180 (John Wileys & Sons Ltd, 2018).

[15] Nielsen LR, Siegismund HR (1999). Interspecific differentiation and hybridization in Vanilla species (Orchidaceae). Heredity.

[16] Besse P (2004). RAPD genetic diversity in cultivated vanilla: Vanilla planifolia, and relationships with V-tahitensis and V-pompona. Plant Sci..

[17] Minoo D (2008). Genetic variations and interrelationships in Vanilla planifolia and few related species as expressed by RAPD polymorphism. Genet. Resourc. Crop Evol..

[18] Schlüter PM, Arenas MAS, Harris SA (2007). Genetic variation in Vanilla planifolia (Orchidaceae). Econ. Bot..

[19] Bory S (2008). Patterns of introduction and diversification of Vanilla planifolia (Orchidaceae) in Reunion Island (Indian Ocean). Am. J. Bot..

[20] Lepers-Andrzejewski S, Causse S, Caromel B, Wong M, Dron M (2012). Genetic linkage map and diversity analysis of Tahitian vanilla (Vanilla × tahitensis, Orchidaceae). Crop Sci..

[21] Ramos-Castellá AL (2017). Evaluation of molecular variability in germplasm of vanilla (Vanilla planifolia G. Jackson in Andrews) in Southeast Mexico: implications for genetic improvement and conservation. Plant Genet. Resourc..

[22] Bory S (2008). Development of microsatellite markers in cultivated vanilla: Polymorphism and transferability to other vanilla species. Sci. Hort..

[23] Perez VB (2016). Molecular and microclimatic characterization of two plantations of Vanilla planifolia (Jacks ex Andrews) with divergent backgrounds of premature fruit abortion. Sci. Hort..

[24] Gigant, R. L. *et al*. Microsatellite markers confirm self-pollination and autogamy in wild populations of Vanilla mexicana Mill.(syn. V. inodora) (Orchidaceae) in the Island of Guadeloupe in *Microsatellite Markers* (ed. Abdurakhmonov, I.) 529–592 (InTech, 2016).

[25] Soto Arenas MA, Dressler RL (2010). A revision of the Mexican and Central American species of Vanilla plumier ex Miller with a characterization of their ITS region of the nuclear ribosomal DNA. Lank. Inter. J. Orchid..

[26] Cameron KM (2004). Utility of plastid psaB gene sequences for investigating intrafamilial relationships within Orchidaceae. Mol. Phyl. Evol..

[27] Bouetard A (2010). Evidence of transoceanic dispersion of the genus Vanilla based on plastid DNA phylogenetic analysis. Mol. Phylo. Evol..

[28] Cameron KM (2006). & Carmen Molina, M. Photosystem II gene sequences of psbB and psbC clarify the phylogenetic position of Vanilla (Vanilloideae, Orchidaceae). Cladistics.

[29] Cameron KM (1999). A phylogenetic analysis of the Orchidaceae: evidence from rbcL nucleotide sequences. Am. J. Bot..

[30] Divakaran M, Babu KN, Ravindran PN, Peter KV (2006). Interspecific hybridization in vanilla and molecular characterization of hybrids and selfed progenies using RAPD and AFLP markers. Sci. Hort..

[31] Verma PC (2009). The extent of genetic diversity among Vanilla species: comparative results for RAPD and ISSR. Ind. Crop Prod..

[32] Sreedhar R, Venkatachalam L, Roohie K, Bhagyalakshmi N (2007). Molecular analyses of Vanilla planifolia cultivated in India using RAPD and ISSR markers. Orchid Sci. Biotech..

[33] Lubinsky P (2008). Neotropical roots of a Polynesian spice: the hybrid origin of Tahitian vanilla, Vanilla tahitensis (Orchidaceae). Amer. J. Bot..

[34] Cameron KM (2009). On the value of nuclear and mitochondrial gene sequences for reconstructing the phylogeny of vanilloid orchids (Vanilloideae, Orchidaceae). Ann. Bot..

[35] Villanueva-Viramontes S (2017). Wild Vanilla planifolia and its relatives in the Mexican Yucatan Peninsula: Systematic analyses with ISSR and ITS. Bot. Sci..

[36] Nissar VM, Hrideek T, Kuruvilla K, Madhusoodanan K, Thomas J (2006). Studies on pollination, inter specific hybridization and fruit development in vanilla. J. Plant. Crop..

[37] Belanger, F. C. & Havkin-Frenkel, D. Molecular analysis of a Vanilla hybrid cultivated in Costa Rica in(eds In *Handbook of Vanilla Science and* Technology (eds Havkin-Frenkel, D. & Belanger F. C.) 256–265 (John Wileys & Sons Ltd, 2018).

[38] Nielsen LR (2000). Natural hybridization between Vanilla claviculata (W. Wright) Sw. and V. barbellata Rchb. f.(Orchidaceae): genetic, morphological, and pollination experimental data. Bot. J. Linn. Soc..

[39] Soto Arenas, M. A. Filogeografía y recursos genéticos de las vainillas de México. *Instituto Chinoin AC. Informe final SNIB-Conabio, proyecto J* **101** (1999).

[40] Lubinsky, P. Conservation of wild *vanilla* in First international congress on the future of Vanilla business. Princeton N.J. Novemeber 11–12 (2003).

[41] Ecott, T. *Vanilla: Travels in search of the Luscious Substance* (Penguin UK, 2005).

[42] Correll DS (1953). Vanilla-Its botany, history, cultivation and economic import. Econ. Bot..

[43] Bouriquet, G. Le vanillier et la vanille dans le monde (Paul Lechevalier, 1954).

[44] Rao X (2014). A deep transcriptomic analysis of pod development in the vanilla orchid (Vanilla planifolia). Bmc Genom..

[45] Simao FA, Waterhouse RM, Ioannidis P, Kriventseva EV, Zdobnov EM (2015). BUSCO: assessing genome assembly and annotation completeness with single-copy orthologs. Bioinformatics.

[46] Pritchard JK, Stephens M, Donnelly P (2000). Inference of population structure using multilocus genotype data. Genetics.

[47] Cameron, K. *Vanilla orchids: Natural history and cultivation* (Timber Press, 2012).

[48] Travnicek P (2015). Challenges of flow-cytometric estimation of nuclear genome size in orchids, a plant group with both whole-genome and progressively partial endoreplication. Cytom Part A.

[49] Lapeyre-Montes, F., Conejero, G., Verdeil, J.-L. & Odoux, E. Anatomy and biochemistry of vanilla bean development in *Vanilla Medicinal and Aromatic Plants - Industrial Profiles* (eds Odoux, E. & Grisoni, M.) 149–172 (CRC Press, 2010).

[50] Brunschwig, C., Collard, F.X., Lepers-Andrzejewski, S. & Raharivelomanana, P. Tahitian Vanilla (Vanilla × tahitensis): A Vanilla Species with unique features in *Active Ingredients from Aromatic and Medicinal Plants* (ed. El-Shemy, H.) Ch. 3; 10.5772/66621 (InTech, 2017).

[51] Gretzinger, N. & Dean, D. Vanilla production in the context of culture, economics, and ecology of Belize in *Handbook of Vanilla Science and* Technology, (eds Havkin-Frenkel, D. & Belanger, F. C.) 50–68 (John Wileys & Sons Ltd, 2018).

[52] Lusher WR, Jackson JL, Morgan KT (2008). The Florida automated weather network: ten years of providing weather information to Florida growers. Proc. Florida St. Hort. Soc..

[53] Aronesty E (2013). Comparison of sequencing utility programs. The Open Bioinformatics Journal.

[54] Schmieder R, Edwards R (2011). Quality control and preprocessing of metagenomic datasets. Bioinformatics.

[55] Liu YC, Schroder J, Schmidt B (2013). Musket: a multistage k-mer spectrum-based error corrector for Illumina sequence data. Bioinformatics.

[56] Li RQ (2010). The sequence and de novo assembly of the giant panda genome. Nature.

[57] Marcais G, Kingsford C (2011). A fast, lock-free approach for efficient parallel counting of occurrences of k-mers. Bioinformatics.

[58] Vurture GW (2017). GenomeScope: fast reference-free genome profiling from short reads. Bioinformatics.

[59] Chikhi R, Rizk G (2013). Space-efficient and exact de Bruijn graph representation based on a Bloom filter. Algorithm Mol. Biol..

[60] Luo RB (2012). SOAPdenovo2: an empirically improved memory-efficient short-read de novo assembler. Gigascience.

[61] Kim D, Landmead B, Salzberg SL (2015). HISAT: a fast spliced aligner with low memory requirements. Nat. Meth..

[62] Glaubitz JC (2014). TASSEL-GBS: A High Capacity Genotyping by Sequencing Analysis Pipeline. Plos One.

[63] Danecek P (2011). The variant call format and VCFtools. Bioinformatics.

[64] Lischer HEL, Excoffier L (2012). PGDSpider: an automated data conversion tool for connecting population genetics and genomics programs. Bioinformatics.

[65] Stamatakis A (2014). RAxML version 8: a tool for phylogenetic analysis and post-analysis of large phylogenies. Bioinformatics.

[66] Evanno G, Regnaut S, Goudet J (2005). Detecting the number of clusters of individuals using the software STRUCTURE: a simulation study. Mol. Ecol..

[67] Earl DA, Vonholdt BM (2012). STRUCTURE HARVESTER: a website and program for visualizing STRUCTURE output and implementing the Evanno method. Conserv. Genet. Resourc..

[68] Zheng XW (2012). A high-performance computing toolset for relatedness and principal component analysis of SNP data. Bioinformatics.

[69] Jombart T, Devillard S, Balloux F (2010). Discriminant analysis of principal components: a new method for the analysis of genetically structured populations. BMC genetics.

[70] Weir BS, Cockerham CC (1984). Estimating F-statistics for the analysis of population-structure. Evol..

[71] Edgar RC (2004). MUSCLE: multiple sequence alignment with high accuracy and high throughput. Nuc. Acid. Res..

