# Supplementary material for: Genomics-based diversity analysis of Vanilla species using a Vanilla planifolia draft genome and Genotyping-By-Sequencing
Source: Sci Rep. 2019 Mar 4;9:3416. doi: 10.1038/s41598-019-40144-1 (PMC6399343; doi:10.1038/s41598-019-40144-1)
Supplement: Supplementary file 1 — Supplementary Information [file 41598_2019_40144_MOESM1_ESM.pdf]

Supplementary Information

Genomics-based diversity analysis of Vanilla species using a Vanilla planifolia draft genome and Genotyping-By-Sequencing

Ying Hu[1], Marcio F. R. Resende Jr.[1], Aureliano Bombarely[2], Maria Brym[3], Elias Bassil[3]*, Alan Chambers[3]*
[1] Horticultural Sciences Department, University of Florida, Gainesville, FL, USA
[2] School of Plant and Environmental Sciences, Virginia Tech, Blacksburg, VA, USA
[3] Tropical Research and Education Center, Horticultural Sciences Department, Homestead, FL, USA

Corresponding Authors
Elias Bassil ebassil@ufl.edu, Alan H. Chambers ac@ufl.edu

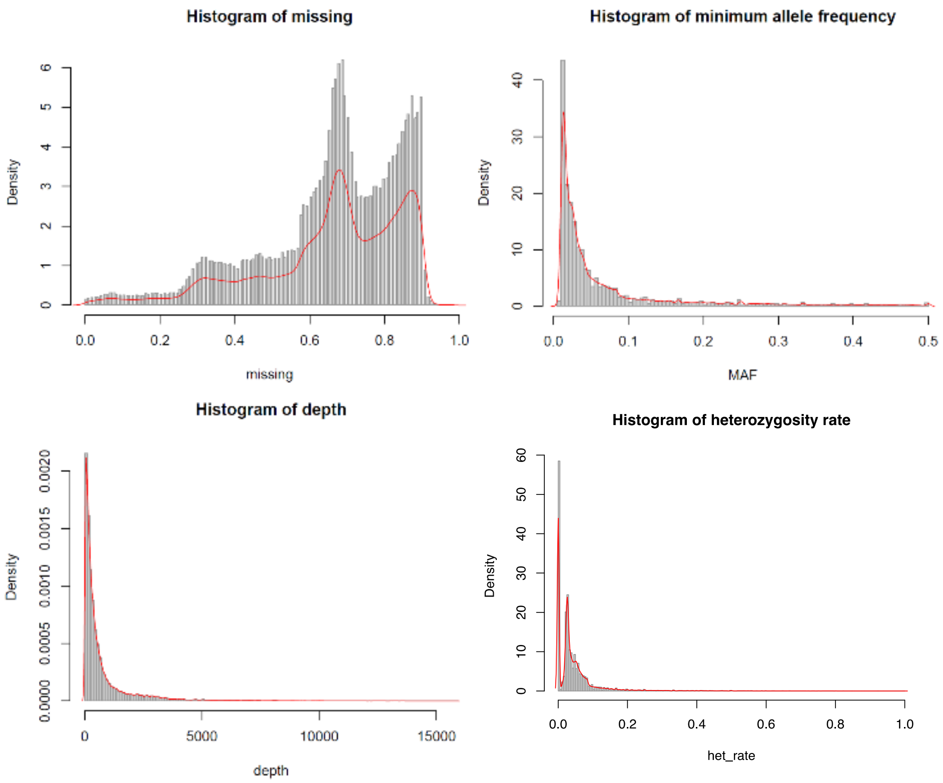

Supplementary Figure S1. Histograms of missing SNPs, minimum allele frequency, SNP mean depth, and heterozygosity rate. Results include 5,082 SNPs filtered from the total 521,732 SNPs by maximum missing rate, minor allele frequency, minimum read depth, maximum read depth, maximum heterozygosity rate, and linkage disequilibrium.

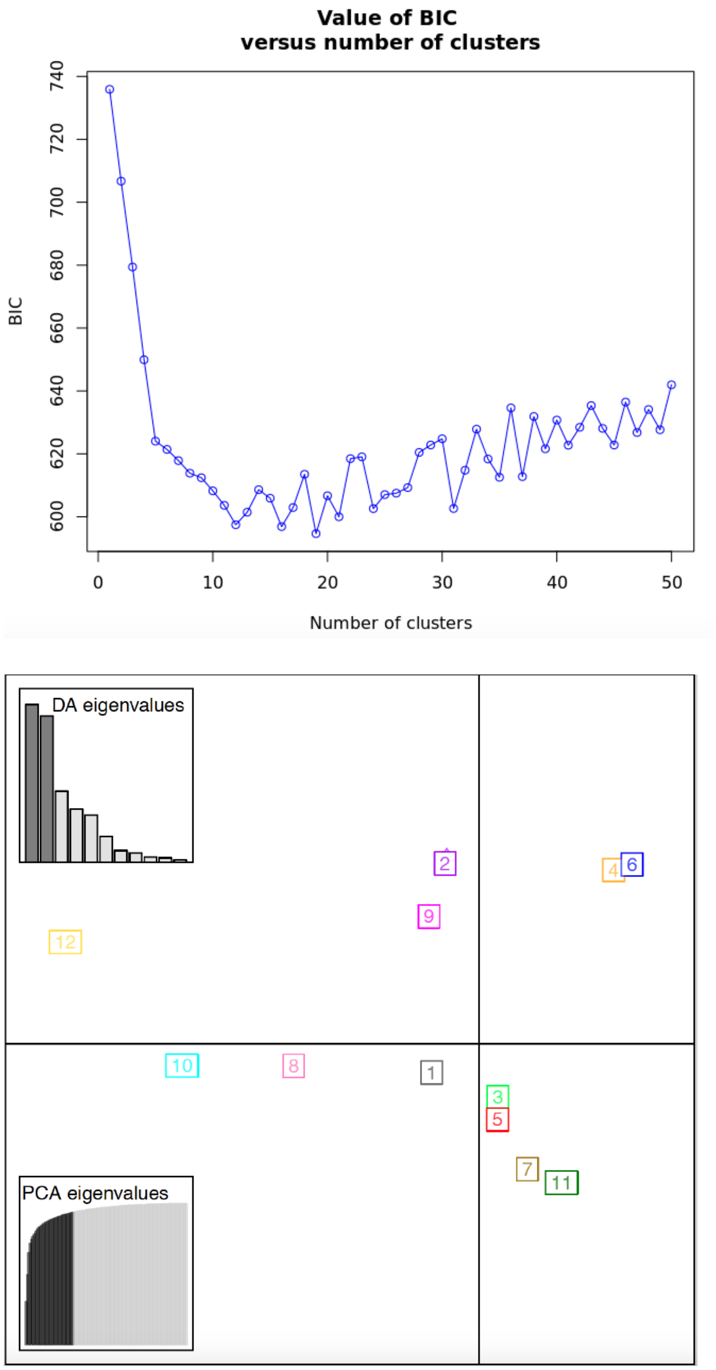

Supplementary Figure S2. Discriminant analysis of principle components (DAPC) on 5,082 filtered SNPs. Results of find.clusters analysis on SNP data identifying optimal number of groups (top). K=12 groups were determined to be optimal based on the lowest value of the Bayesian information criterion (BIC). Results of DAPC analysis on SNP data using the optimal groups identified by find.clusters (bottom).

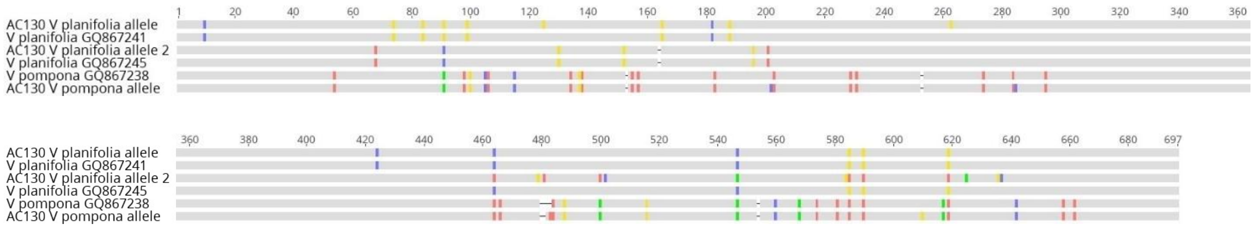

Figure S3. ITS sequence alignment for representative AC130, *V. planifolia*, and *V. po*mpona alleles. AC130 was confirmed as a hybrid by comparing cloned ITS sequences from this accession to published ITS sequences from NCBI. Colored bars represent SNP variants among sequences.

Supplementary Table S1. Missing rate (F_Miss), mean read depth (MEAN_DEPTH), heterozygosity rate (F_het), total read counts (Total_readcounts), number of mapped reads (Mapped_readcounts), and mapping rate (number of mapped reads divided by the total number of reads, F_mapped) for each accession in this study (INDV).

INDV	F_MISS	MEAN_DEPTH	F_het	Total_readcounts	Mapped_readcounts	F_mapped
AC101	0.6678	7.6552	0.0444	1702711	1095698	0.6435
AC102	0.8849	2.7756	0.0313	1273411	1193469	0.9372
AC103	0.6363	6.4555	0.0631	1002741	935938	0.9334
AC104	0.6541	6.8092	0.0584	1021392	962785	0.9426
AC105	0.8320	2.5105	0.0655	859321	802578	0.9340
AC106	0.4510	7.5754	0.0457	709439	651374	0.9182
AC107	0.6262	5.7669	0.0600	826420	778007	0.9414
AC108	0.3954	8.2830	0.0401	960858	905497	0.9424
AC109	0.4917	11.7423	0.0498	1382589	1298892	0.9395
AC110	0.6193	9.2317	0.0627	1509176	1406870	0.9322
AC111	0.8885	3.1522	0.0274	1479631	1383117	0.9348
AC112	0.9003	2.3357	0.0348	1018451	963000	0.9456
AC113	0.4050	12.6263	0.0363	1283603	1207835	0.9410
AC114	0.7100	7.9936	0.0331	1261750	1185601	0.9396
AC115	0.8445	3.4330	0.0712	1208126	1132419	0.9373
AC116	0.6857	7.2913	0.0404	1169986	1102663	0.9425
AC117	0.3912	15.3937	0.0349	1689264	1405652	0.8321
AC118	0.8675	2.8981	0.0631	1100658	1031003	0.9367
AC119	0.8653	4.1325	0.0409	1563129	1397531	0.8941
AC120	0.4318	10.1431	0.0322	1571829	1417990	0.9021
AC121	0.8409	3.8244	0.0658	1351988	1272727	0.9414
AC122	0.8478	3.1805	0.0699	1132766	1059960	0.9357
AC123	0.8110	3.7772	0.0405	1415925	1333599	0.9419
AC124	0.6679	9.1970	0.0433	1569450	1405471	0.8955
AC125	0.8753	3.0327	0.0530	1261126	1190211	0.9438
AC126	0.4015	13.4265	0.0346	1403526	1316542	0.9380
AC127	0.8677	3.3031	0.0500	1217511	1146952	0.9420
AC128	0.9019	2.7442	0.0370	1237361	1171302	0.9466
AC129	0.6777	7.3809	0.0430	1207540	1137128	0.9417
AC130	0.3325	11.6410	0.0543	1245253	1164765	0.9354
AC131	0.8405	3.6244	0.0686	1217584	1145359	0.9407
AC132	0.5497	8.5513	0.0788	1186054	1114081	0.9393
AC133	0.4127	13.5738	0.0363	1161176	1023830	0.8817
AC134	0.8498	3.3279	0.0673	1204291	1123762	0.9331
AC135	0.6950	7.3267	0.0373	1127332	1059853	0.9401
AC136	0.7049	6.9328	0.0368	979223	922408	0.9420
AC137	0.6953	7.4591	0.0393	1228976	1159039	0.9431
AC138	0.6999	6.9776	0.0409	1080744	1015826	0.9399
AC139	0.8715	3.7851	0.0428	1313563	1238533	0.9429

AC140	0.8758	3.1817	0.0422	1124344	1044480	0.9290
AC141	0.8942	3.0878	0.0322	1382801	1302187	0.9417
AC142	0.6835	7.6019	0.0423	1282825	1208402	0.9420
AC143	0.7847	5.9457	0.0332	1475289	1381289	0.9363
AC144	0.8730	3.1817	0.0562	1272049	1197605	0.9415
AC145	0.8707	2.7137	0.0517	928049	872551	0.9402
AC146	0.8694	3.2929	0.0529	1183059	1113428	0.9411
AC147	0.5870	7.9652	0.0557	1461253	1374415	0.9406
AC148	0.9051	2.5050	0.0288	1012188	959139	0.9476
AC149	0.8668	2.9622	0.0503	1020096	959147	0.9403
AC150	0.8668	2.9072	0.0454	1011492	942778	0.9321
AC151	0.4359	10.5502	0.0390	1198373	1115532	0.9309
AC152	0.8726	3.0584	0.0459	998069	943661	0.9455
AC153	0.6581	5.9399	0.0584	885450	832549	0.9403
AC154	0.9080	1.5053	0.0358	798370	742886	0.9305
AC155	0.7470	6.4023	0.0315	904735	848722	0.9381
AC156	0.6659	5.5706	0.0557	873839	824535	0.9436
AC157	0.5371	9.5282	0.0823	1400060	1315257	0.9394
AC158	0.7039	5.9184	0.0454	983622	926992	0.9424
AC159	0.6341	7.4923	0.0638	1197069	1125203	0.9400
AC160	0.4378	9.3481	0.0397	1095125	1019003	0.9305
AC161	0.9109	2.3586	0.0307	1021470	961955	0.9417
AC162	0.8892	1.1285	0.0768	424993	384769	0.9054
AC164	0.4527	9.7346	0.0362	1121629	1046816	0.9333
AC165	0.7070	5.9426	0.0393	1158956	1096692	0.9463
AC166	0.6364	7.8215	0.0573	1347035	1264177	0.9385
AC167	0.6452	6.7758	0.0569	1393073	1310508	0.9407
AC168	0.4220	9.2387	0.0352	1229386	1137917	0.9256
AC169	0.6499	6.1902	0.0570	1225243	1140834	0.9311
AC170	0.5271	7.5764	0.0502	1148501	1070933	0.9325
AC171	0.4279	9.2183	0.0357	1232666	1138693	0.9238
AC172	0.8688	2.9915	0.0640	1225529	1153053	0.9409
AC173	0.4866	7.1756	0.0360	1016347	940776	0.9256
AC174	0.4381	9.8647	0.0385	1109863	1023604	0.9223
AC175	0.6778	4.0242	0.0612	854786	792439	0.9271
AC176	0.4451	8.2828	0.0401	1019600	943506	0.9254
AC177	0.7656	2.9367	0.0342	729856	680633	0.9326
AC178	0.4946	5.4575	0.0381	791307	731328	0.9242
AC179	0.7003	5.2425	0.0492	922327	860827	0.9333
AC180	0.4464	8.2473	0.0401	926877	859723	0.9275
AC181	0.4369	8.0271	0.0361	1016258	952139	0.9369
AC182	0.8246	2.6473	0.0598	1037109	977925	0.9429
AC183	0.6474	6.3294	0.0553	1100825	1033886	0.9392
AC184	0.4183	10.1539	0.0365	1049946	977108	0.9306

AC185	0.5128	10.2530	0.0368	1106923	1035680	0.9356
AC186	0.4203	9.4675	0.0405	1033508	962323	0.9311
AC187	0.4499	7.9518	0.0378	1063769	997294	0.9375
AC188	0.4310	9.0943	0.0354	1115975	1050555	0.9414
AC189	0.5597	7.7200	0.0781	1692299	1098659	0.6492
AC190	0.6361	7.6234	0.0574	1526647	1103977	0.7231
AC191	0.9422	0.9872	0.0375	1670433	1117493	0.6690
AC192	0.9413	0.9378	0.0390	1402635	1055131	0.7522
AC193	0.8741	2.8065	0.0486	1395910	930068	0.6663
AC194	0.5070	10.2593	0.0517	1357813	1011516	0.7450
AC195	0.4201	9.9625	0.0343	1090002	1024664	0.9401
AC196	0.4442	7.7727	0.0331	872417	820701	0.9407
AC197	0.4425	7.4127	0.0342	908257	853074	0.9392
AC198	0.4131	11.5246	0.0342	1040117	975499	0.9379
AC199	0.4230	10.3785	0.0398	980781	915374	0.9333
AC200	0.6621	4.8250	0.0643	721578	669181	0.9274
AC201	0.4271	9.4882	0.0365	1034226	972673	0.9405
AC202	0.4445	9.8416	0.0397	1142537	1059420	0.9273
AC203	0.4526	9.5420	0.0383	1123601	1050136	0.9346
AC204	0.7398	5.9326	0.0376	990312	923119	0.9321
AC205	0.6922	7.3813	0.0389	891063	821829	0.9223
AC206	0.6327	10.0804	0.0367	1141217	1060179	0.9290
AC207	0.7138	6.9602	0.0426	1088146	1014708	0.9325
AC208	0.7371	6.9657	0.0305	1090027	1026024	0.9413
AC209	0.4213	10.7187	0.0364	1155495	1086302	0.9401
AC210	0.7898	5.2000	0.0398	925209	862141	0.9318
AC211	0.7069	8.3476	0.0299	1244635	1166519	0.9372
AC212	0.7482	6.4364	0.0328	1005105	940494	0.9357
AC213	0.7254	5.7215	0.0369	843790	793825	0.9408
AC214	0.7199	6.5414	0.0507	1192704	1121148	0.9400
AC215	0.7226	6.5732	0.0513	1275902	1202583	0.9425
AC216	0.7122	7.5981	0.0502	1338870	1263259	0.9435
AC217	0.7194	6.4827	0.0496	1171653	1107048	0.9449
AC218	0.4312	10.6667	0.0346	1255344	1185221	0.9441
AC219	0.4098	12.8305	0.0345	1080298	1018860	0.9431

Supplementary Table S2. Allele calls for each accession in this study for the 5,082 SNPs. Contig_position is the contig and base pair position of the SNP when mapping onto the VaplaK095A02V draft assembly. SNP values 0, 1, 2, or NA represent the number of reference alleles where 0 = homozygous alternate allele, 1 = heterozygous, 2 = homozygous reference allele, and NA = missing data.

Supplementary Table S3. Accession numbers, species assignment, gene target, and GenBank ID for single gene sequences from this study.

Accession	Species	Gene Target	GenBank
AC101	V. phaeantha	rbcL	MK201688
AC104	V. pompona	rbcL	MK201689
AC106	V. planifolia	rbcL	MK201690
AC108	V. planifolia and unknown	rbcL	MK201691
AC109	V. planifolia x V. phaeantha	rbcL	MK201692
AC110	V. pompona	rbcL	MK201693
AC115	V. dilloniana	rbcL	MK201694
AC118	V. imperialis	rbcL	MK201695
AC119	V. poitaei	rbcL	MK201696
AC123	V. appendiculata	rbcL	MK201697
AC124	V. phaeantha	rbcL	MK201698
AC126	V. planifolia	rbcL	MK201699
AC127	V. roscheri	rbcL	MK201700
AC129	V. phaeanthan and unknown	rbcL	MK201701
AC130	V. planifolia x V. pompona	rbcL	MK201702
AC132	V. pompona x V. phaeantha	rbcL	MK201703
AC133	V. planifolia	rbcL	MK201704
AC134	V. dilloniana	rbcL	MK201705
AC139	V. barbellata	rbcL	MK201706
AC142	V. pompona and V. odorata	rbcL	MK201707
AC147	V. phaeantha x V. pompona	rbcL	MK201708
AC158	V. pompona and V. odorata	rbcL	MK201709
AC165	V. pompona and V. odorata	rbcL	MK201710
AC168	V. planifolia	rbcL	MK201711
AC170	V. planifolia x V. phaeantha	rbcL	MK201712
AC173	V. planifolia	rbcL	MK201713
AC174	V. planifolia	rbcL	MK201714
AC175	V. pompona and V. odorata	rbcL	MK201715
AC177	V. odorata	rbcL	MK201716
AC178	V. planifolia	rbcL	MK201717
AC181	V. planifolia	rbcL	MK201718
AC183	V. pompona	rbcL	MK201719
AC184	V. planifolia	rbcL	MK201720
AC185	V. planifolia	rbcL	MK201721
AC186	V. planifolia	rbcL	MK201722
AC191	V. mexicana	rbcL	MK201723
AC193	V. barbellata	rbcL	MK201724
AC194	V. planifolia x V. phaeantha	rbcL	MK201725
AC195	V. planifolia	rbcL	MK201726
AC196	V. planifolia	rbcL	MK201727

AC197	V. planifolia	rbcL	MK201728
AC202	V. planifolia	rbcL	MK201729
AC203	V. planifolia	rbcL	MK201730
AC204	V. hartii	rbcL	MK201731
AC205	V. x tahitensis	rbcL	MK201732
AC206	V. x tahitensis	rbcL	MK201733
AC207	V. odorata	rbcL	MK201734
AC208	V. ensifolia	rbcL	MK201735
AC209	V. planifolia	rbcL	MK201736
AC210	V. odorata	rbcL	MK201737
AC211	V. odorata and unknown	rbcL	MK201738
AC212	V. ensifolia	rbcL	MK201739
AC213	V. odorata	rbcL	MK201740
AC214	V. palmarum	rbcL	MK201741
AC215	V. palmarum	rbcL	MK201742
AC216	V. palmarum	rbcL	MK201743
AC217	V. palmarum	rbcL	MK201744
AC130	V. planifolia x V. pompona	ITS - planifolia allele 1	MK190714
AC130	V. planifolia x V. pompona	ITS - planifolia allele 2	MK190715
AC130	V. planifolia x V. pompona	ITS - pompona allele	MK190716

Supplementary Data S1. Species-specific SNPs screened from the total 521,732 SNPs identified in this study. Contig_position identifies the SNP called when mapping onto the VaplaK095A02V draft assembly, the alleles column is the reference and alternate allele, chrom is the contig number, pos is the bp location of the SNP in the contig. All accessions are labeled with their corresponding alleles by columns. Each tab in the database contains putative species-specific alleles for that species.
